# Functional inhibition of lactate dehydrogenase suppresses pancreatic adenocarcinoma progression

**DOI:** 10.1002/ctm2.467

**Published:** 2021-06-28

**Authors:** Chien‐shan Cheng, Hor‐Yue Tan, Ning Wang, Lianyu Chen, Zhiqiang Meng, Zhen Chen, Yibin Feng

**Affiliations:** ^1^ Department of Integrative Oncology Fudan University Shanghai Cancer Center Shanghai China; ^2^ Department of Oncology, Shanghai Medical College Fudan University Shanghai China; ^3^ Li Ka Shing Faculty of Medicine School of Chinese Medicine, The University of Hong Kong Hong Kong China

**Keywords:** AMPK/mTOR pathway, berberine, L‐lactate, lactate dehydrogenase, pancreatic adenocarcinoma

## Abstract

**Background:**

Pancreatic adenocarcinoma (PAAD) a highly lethal malignancy. The current use of clinical parameters may not accurately predict the clinical outcome, which further renders the unsatisfactory therapeutic outcome.

**Methods:**

In this study, we retrospectively analyzed the clinical‐pathological characteristics and prognosis of 253 PAAD patients. Univariate, multivariate, and Kaplan‐Meier survival analyses were conducted to assess risk factors and clinical outcomes. For functional study, we performed bidirectional genetic manipulation of lactate dehydrogenase A (LDHA) in PAAD cell lines to measure PAAD progression by both in vitro and in vivo assays.

**Results:**

LDHA is particularly overexpressed in PAAD tissues and elevated serum LDHA‐transcribed isoenzymes‐5 (LDH‐5) was associated with poorer patients’ clinical outcomes. Genetic overexpression of LDHA promoted the proliferation and invasion in vitro, and tumor growth and metastasis in vivo in murine PAAD orthotopic models, while knockdown of LDHA exhibited opposite effects. LDHA‐induced L‐lactate production was responsible for the LDHA‐facilitated PAAD progression. Mechanistically, LDHA overexpression reduced the phosphorylation of metabolic regulator AMPK and promoted the downstream mTOR phosphorylation in PAAD cells. Inhibition of mTOR repressed the LDHA‐induced proliferation and invasion. A natural product berberine was selected as functional inhibitor of LDHA, which reduced activity and expression of the protein in PAAD cells. Berberine inhibited PAAD cells proliferation and invasion in vitro, and suppressed tumor progression in vivo. The restoration of LDHA attenuated the suppressive effect of berberine on PAAD.

**Conclusions:**

Our findings suggest that LDHA may be a novel biomarker and potential therapeutic target of human PAAD.

## Graphical abstract

LDHA‐transcribed isoenzymes‐5 is a novel serum prognostic biomarker in PAAD

LDHA overexpression is positively correlated with PAAD progression

LDHA and L‐lactate upregulation suppressed AMPK activation and inhibition of mTOR

Berberine acts as a LDHA functional inhibitor, suppresses LDHA and L‐lactate and thereby represses PAAD progression

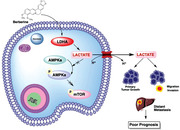

**Abbreviations**
AMP: Adenosine monophosphate
AMPK: AMP‐activated protein kinase
AST: Aspartate transaminase
ATP: Adenosine triphosphate
BBR: Berberine
BMI: Body mass index
Ca19‐9: Carbohydrate antigen19‐9
CM: Chinese medicine
CTL: Control
H&E: Hematoxylin and eosin
IC50: Half‐maximal inhibitory concentration
IHC: Immunohistochemistry
LDH: Lactate dehydrogenase
LDH‐5: LDHA‐transcribed isoenzymes‐5
LDHA: Lactate dehydrogenase A
LDHB: Lactate dehydrogenase B
MCT: Monocarboxylate transporter
mTOR: Mammalian target of rapamycin
NADH: Nicotinamide adenine dinucleotide
PAAD: Pancreatic adenocarcinoma
SPR: Surface plasmon resonance

## 1 BACKGROUND

Pancreatic adenocarcinoma (PAAD) is a highly malignant disease ranking as the fourth leading cause of cancer‐related mortality globally, with a 5‐year survival of less than 5%.^1^ Despite efforts on improving treatment modalities, PAAD remains a major clinical challenge, especially for patients with advanced disease.^2^, ^3^ The routinely screened clinical parameters cannot accurately predict the patients’ clinical outcomes, especially those with advanced diseases, their potential response to treatments, or tumor behaviors. Therefore, there is an urgent need to identify novel biological markers for a more accurate prediction of the disease's natural history. In addition, deepening the understanding of the molecular mechanisms of disease progression and determining effective targets for the treatment of PAAD are of great significance for improving the clinical outcome of the disease.

Lactate dehydrogenase (LDH) is a tetramer enzyme that regulates the final step of aerobic glycolysis and is associated with metabolic adaptation to meet the energetic and biosynthetic demands of malignancies.^4^, ^5^, ^6^, ^7^ LDH consists of two common polypeptide subunits: the M‐subunit (LDHA encoded) and the H‐subunit (LDHB encoded).^7^ As a result of the different subunits, five isoforms (LDH‐1 to LDH‐5) have been identified.^7^ In PAAD patients, consistent with previous studies,^8^, ^9^ we have demonstrated that lactate dehydrogenase elevation is an independent unfavorable prognostic factor.^10^ However, the prevailing types of LDH and their expressions in PAAD is yet to be characterized.

This study investigated the prognostic role of LDH isoenzymes by retrospective analyses. By employing isoenzyme electrophoresis, we identified that serum LDHA‐transcribed isoenzymes‐5 (LDH‐5) level is negatively associated with patients’ overall survival, prognosis, tumor aggressiveness, and maybe a novel serum biomarker. Using in vitro and in vivo methods, we explored the tumor‐promoting role of the LDHA gene in PAAD. Mechanistically, the elevated LDHA expression and enhanced L‐lactate production fuelled pancreatic cancer growth and progression, and the activity depends on the AMP‐activated protein kinase (AMPK)/ mammalian target of rapamycin (mTOR) signaling pathway. Moreover, in seeking an effective targeted‐therapy for PAAD patients’ management, our study later identified a small molecule, berberine, which functionally inhibits LDHA and suppresses in vitro and in vivo PAAD progression. Experimental findings postulate the role of LDHA overexpression‐induced L‐lactate signaling and its interplay with AMPK/mTOR signaling, which may provide scientific evidence for future precision therapy of PAAD.

## 2 METHODS

### 2.1 Patients

A total of 253 patients who were treatment‐naive and pathologically diagnosed with pancreatic adenocarcinoma between January 2013 and December 2015 at the Department of Integrative Oncology, Fudan University Shanghai Cancer Center were included. The patients’ clinical data, including gender, age, BMI (body mass index), tumor stage at diagnosis, tumor location, maximum tumor diameter, serum Carbohydrate antigen19‐9 (Ca19‐9) level, serum lactate dehydrogenase (LDH) level, the ratio of aspartate transaminase (AST) and alanine aminotransferase (ALT) (AST/ALT ratio), presence of metastases (liver, lung, bone, and retroperitoneal lymph node) at diagnosis, treatment received (chemotherapy, ablation therapy, and Chinese Medicine [CM] treatment) were obtained from electrical medical records. All patients were subjected to follow‐ups for at least 12 months. Serum samples were collected upon initial admission and stored at −80°C for further analyses. The ethics committee of the Department of Oncology, Fudan University approved the protocol (Ref. No. 050432‐4‐1212B) and written informed consent obtained from individual patients according to institutional guidelines. All procedures were performed following institutional standards, guidelines, and with the 1964 Helsinki declaration and its later amendments.

### 2.2 Bioinformatics study

#### 2.2.1 Oncomine database analysis

The genome expression of LDHA in pancreatic cancer was mined in the Oncomine database,^11^ and five datasets (Logsdon Pancreas, Segara Pancreas, Buchholz Pancreas, Pei Pancreas, and Badea Pancreas) were used to validate the mRNA expression of LDHA. The transcriptional expression of LDHA of the cancer tissue and their corresponding adjacent noncancerous pancreatic tissue were obtained from the Oncomine database and compared by Student's *t*‐test. The thresholds employed are as followings: *P* = 0.01; fold‐change = 1.5; gene ranking = 10%.

#### 2.2.2 The cancer genome atlas (TCGA) and gene expression omnibus (GEO) database analyses

To compare the gene expression profile across all adenocarcinoma samples and paired normal tissues, the Gene Expression Profiling Interactive Analysis (GEPIA) (http://gepia.cancer‐pku.cn/index.html),^12^ which is an online database including samples from TCGA and the GTEx projects, was used (Log_2_FC Cutoff: 2; *q*‐value Cutoff: 0.01). The relative transcriptional expression of LDHA between tumor and normal samples obtained from Gene Expression GEPIA was also used to generate overall survival (OS) and disease‐free survival (DFS) curves with a log‐rank test based on median LDHA expression. The log‐rank *P*, the hazard ratio (HR), and 95% confidence intervals (CI) were computed. GEO datasets (GDS4103 and GDS4336) were compared by Student's *t*‐test. Statistical significant difference was considered when a *P* < 0.01.

#### 2.2.3 The cancer cell line encyclopaedia analysis

The mRNA expression of LDHA in pancreatic cancer cell lines was retrieved from the Cancer Cell Line Encyclopaedia (http://portals.broadinstitute.org/ccle/)^13^ to verify the mRNA expression level in pancreatic cancer cell lines.

### 2.3 Prediction of small molecule interaction

The in silico molecular docking model for LDHA in complex with berberine was constructed using the online docking webserver SwissDock (http://www.swissdock.ch/).^14^ The target structure of human LDHA (PDB ID:1I10) was identified from the Protein Data Bank (PDB).^15^ The chemical structure of berberine (PubChem: 12456, ZINC ID: 3779067) was obtained from the ZINC molecule database (http://zinc.docking.org/).^16^ Results of the SwissDock were visualized by UCSF Chimera.^17^

### 2.4 Cell lines and culture conditions

Pancreatic cancer cell lines Panc1, MiaPaCa2, BxPC3, Capan‐2 (Human origin), and Panc02 (Mouse C57BL/6 origin) were obtained from American Type Culture Collection (ATCC; Manassas, VA, USA) and Frederick National Laboratory for Cancer Research (Frederick, MD, USA), respectively. The Panc1, MiaPaCa2, and Panc02 cells were cultured in Dulbecco's Modified Eagle Medium (DMEM, 4.5 g/L glucose; Gibco, Rockville, MD, USA) supplemented with 10% heat‐inactivated fetal bovine serum (FBS; Gibco), 1 mM sodium pyruvate (Cat. P5280, Sigma‐Aldrich, St. Louise, MO, USA), 100 U/mL penicillin (Gibco) and 100 mg/mL streptomycin (Gibco). BxPC3 and Capan‐2 were cultured in RPMI‐1640 medium (Gibco) and McCoy's 5A Medium (Gibco), respectively, supplemented with 10% FBS (Gibco). All cells were maintained in a humidified incubator containing 5% CO_2_ at 37°C.

Berberine chloride (C20H18ClNO4, PubChem CID: 12456) was purchased from Sigma‐Aldrich (Cat. B3251‐10G, Lot: #SLBG1303). For AMPK inhibitor and mTOR inhibitor treatment, cells are treated with compound c (20 μΜ; PubChem CID: 11524144, Sigma‐Aldrich), Rapamycin (10 nM; PubChem CID: 5284616, Cat. R0395, Sigma‐Aldrich), 7ACC2 (10 μΜ; PubChem CID: 72696735, Cat. HY‐D0713, MedChemExpress), respectively, for 24 hours. For lactate treatment, cells are treated with L‐lactate (10 mM; PubChem CID: 107689, Cat. L1750, Sigma‐Aldrich) for 48 hours.

### 2.5 Plasmids, short hairpin RNA (shRNA), CRISPR/Cas9 gene editing and transfection

Lentiviral vectors for the mouse LDHA(GenBank ID NM_001136069.2) cDNA sequence, and LDHA specific RNAi interference sequence were obtained from Hanyin Co. (Shanghai, China). To obtain the stable cell line, Panc02 cells were infected with the lentivirus. Seventy‐two hours after infection, puromycin (2 μg/mL; Thermo Scientific, Waltham, MA, USA) was added for 7 days and the LDHA expression of stably transfected cell lines was verified by western blot.

*LDHA* CRISPR/Cas9 activation and knockdown plasmids (sc‐400403; Santa Cruz Ltd., Dallas, CA, USA) were obtained from Santa Cruz Biotechnology. Transfection of plasmids into Panc‐1 cells as performed using the transfection reagent Lipofectamine™ 2000 (Invitrogen, Carlsbad, CA, USA). Panc‐1 cells treated with transfection reagent alone was included as a mock control. To obtain stable cell line, puromycin (1 μg/mL; Thermo Scientific, Waltham, MA, USA) was added for 7 days and the LDHA expression of stably transfected cell lines was verified by western blot.

### 2.6 Cell viability and clonogenic assay

Five‐thousand cells were seeded in each well of the 96‐well plates and allowed to attach overnight. A series concentrations of berberine were added to the cells to incubate for 24, 48, and 72 hours. Ten microliters of the 3‐(4,5‐dimethylthiazol‐2‐yl)‐2,5‐diphenyltetrazoli‐um bromide (MTT; 5 mg/mL, Sigma‐Aldrich) solution was added as a quantitative colorimetric assay dye four hours before the end of treatment and incubated at 37°C for crystallizations. 200 μL of dimethylsulfoxide (DMSO, Sigma‐Aldrich) was then added to each well to resolve the crystal following removing the supernatant. Absorbance was measured at 540 nm using a Model 680 microplate reader (Bio‐Rad, Hercules, CA, USA).

Clonogenic assay was conducted as previously described^18^ with minor modifications. In brief, 1000 cells were inoculated in each well of the 6‐well plates. Twenty‐four hours after seeding, culture medium was replaced with fresh medium containing indicated concentrations of desired agents and allowed to grow for 10 days. Fresh medium and desired agents were replaced every other day. By the end of the experiment, the cells were fixed in 4% paraformaldehyde (PFA; Sigma‐Aldrich) for 30 minutes and followed by a 2‐hour staining with crystal violet (Sigma‐Aldrich) at room temperature for visualizations. Images were captured under the Chemidoc Imaging system (Bio‐rad, Hercules, CA, USA). The number of colonies formed was counted.

### 2.7 Wound healing migration assay and Boyden chamber invasion assay

To measure the migration capabilities of pancreatic cancer cells, single later of cells were cultured in 6‐well plates until 100% confluency followed by a 6‐hour starvation with serum‐free medium. Afterward, the cell layer was scrapped with a 10 μL pipet tip to create a wound. Drugs at appropriate concentration were added, and images were captured with the EVOS XL Core Cell Imaging System (Life Technologies, Carlsbad, CA, USA) at 0, 24, and 48 hours to observe the rate of wound closure by cell migration.

To measure the invasion capabilities of cells, cultured cells are suspended at a density of 2 × 10^5^ cells/mL in serum‐free medium, and 100 μL of cell suspension with appropriate drug concentration were seeded into the upper chamber of the Transwell (8.0 μm pore size, No. 3464; Corning, Corning, NY, USA). Eight hundred microliters of medium with 10% FBS with appropriate drug concentration was added into the lower chamber of the Transwell. Twenty‐four hours after cell seeding, the cells on the upper surface of the chamber were gently removed using a cotton swab with ice‐cold PBS. Cells attached at the basolateral membrane of the chamber insert were then fixed with 4% PFA (Sigma‐Aldrich) for 30 minutes followed by a 2‐hour staining with crystal violet (Sigma‐Aldrich) at room temperature. Images were captured under microscope, and the invaded cells were quantified by counting five fields at a magnification of ×200 (Leica Microsystems Digital Imaging, Wetzlar, Germany).

### 2.8 In vitro LDH release assay

The LDH release is measured by the Lactic Dehydrogenase based In Vitro Toxicology Assay Kit (Cat. TOX7, Sigma‐Aldrich) according to the manufacturer's instructions. In brief, the supernatant was collected and centrifuged at 250 × *g* for 5 minutes to pellet cells. The supernatant was then incubated for 20‐30 minutes with assay mixtures at 1:2 ratio at room temperature protected from light. The reaction was terminated by adding 1:10 (v/v) of 1N hydrogen chloride (HCl). The absorbance was measured at a wavelength of 490 nm using a microplate reader. The relative LDH release was calculated after normalization by cell number.

### 2.9 Lactate assay

Pancreatic cancer Panc‐1 and Panc02 cells (1 × 10^6^), pancreatic tumor tissue (approximate 1 mm^3^) and serum were prepared for lactate assays. According to the manufacturer's instructions, the L‐lactate amount from the lysates of cells and culture media are measured by the lactate colorimetric assay kit II (K627, BioVision, Milpitas, CA, USA). The absorbance was measured using a microplate reader at a wavelength of 450 nm. The relative lactate intracellular and extracellular lactate content was calculated after normalization by cell number, while the calculation of the relative tumor lactate content was calculated after normalization by protein concentration.

### 2.10 LDHA inhibitor screening assay

The LDHA inhibitor screening assay is measured by the Lactate Dehydrogenase A Inhibitor Colorimetric Screening Kit (K492, BioVision, Milpitas, CA, USA) according to the manufacturer's instructions. Inhibitors including FX‐11(HY‐16214), GSK2837808A(HY‐100681), and Gossypol(HY17510) obtained from the MedChemExpress (MCE, Monmouth Junction, NJ, USA). The absorbance was measured using a microplate reader at a wavelength of 450 nm.

### 2.11 Measurement of mitochondrial membrane potential

The fluorescent, lipophilic, and cationic probe, JC‐1 (C2006, Beyotime, Shanghai, China), was employed to measure the mitochondrial membrane potential (Δψm) according to the manufacturer's directions. Briefly, after indicated treatments, cells were incubated with JC‐1 staining solution and subjected to analysis by a flow cytometer (Canto II, BD Biosciences, San Jose, CA, USA).

### 2.12 Immunoblotting analysis

Cell lysates were prepared by lysing pancreatic cancer cells in RIPA lysis and extraction buffer (Thermo Fisher Scientific Inc., Waltham, MA USA) supplemented with a proteinase inhibitor cocktail (Thermo Fisher Scientific Inc.) and phosphatase inhibitor (Cell Signaling Technology, CST, Danvers, MA, USA). Equal amounts of denatured protein were subjected to sodium dodecyl sulfate polyacrylamide gel electrophoresis (SDS‐PAGE; Bio‐Rad, Hercules, CA) followed by electro‐transferred onto polyvinylidene fluoride (PVDF) membrane (Millipore, Burlington, MA, USA). Membranes were incubated with blocking buffer containing 5% bovine serum albumin (BSA; Sigma‐Aldrich) for 2 hours before overnight incubation with the relevant antibodies: LDHA (1: 1000, ab101562, Abcam), AMPKα (D5A2) (1:1000, CST), Phospho‐AMPKα (Thr172) (40H9) (1:1000, CST), mTOR (7C10) (1:1000, CST), Phospho‐mTOR (Ser2448) (D9C2) (1:1000, CST), β‐Actin (D6A8) (1: 1000, CST). The detailed reactivity of each antibody used are included in the supplementary materials (Table S1). HRP‐conjugated secondary antibody (1: 5000, CST) was applied to the membrane for 2 hours at room temperature, and were then visualized using the Amersham™ ECL Select™ western blotting detection reagent (GE Healthcare, Little Chalfont, Buckinghamshire, UK) using a chemiluminescence imaging system (Bio‐Rad, Hercules, CA, USA).

### 2.13 Reverse transcription‐polymerase chain reaction

Total RNA was extracted by Trizol Reagent (Takara, Shiga, Japan) and the first strand synthesis kit (Takara, Shiga, Japan) was used for reverse‐transcription reaction to prepare cDNA samples. The quantitative real‐time PCR (qRT‐PCR) was conducted by SYBR Green reagents (Takara, Shiga, Japan) on LightCycler 480 real‐time PCR system (Roche Life Science, Basel, Switzerland). The expression of *beta‐actin* was used as internal control for the normalization of targeted genes’ expression. The custom‐made primers (Life Technologies, Carlsbad, CA, USA) for RT‐PCR analysis are as follows: LDHA (h, human) forward: CCCCAGAATAAGATTACAGTTGTTG, LDHA(h) reverse: GAGCAAGTTCATCTGCCAAGTC; LDHA(m, mouse) forward: CTGGCTCCAGTGTGTACGTC, and LDHA(m) reverse: TGGGTGGTTGGTTCCATCAT; β‐actin(h) forward: CTACGTCGCCCTGGACTTCGAGC; and β‐actin(h) reverse: GATGGAGCCGCCGATCCACACGG; and β‐actin(m) forward:  TGTCCACCTTCCAGCAGATGT, β‐actin(m) reverse: AGCTCAGTA ACAGTCCGCCTAG.

### 2.14 Measurement of serum LDH isoenzyme

Serum LDH isoenzymes are measured by QuickGel LD Isoenzyme Kit (No. 3538T; Helena Laboratories Beaumont, TX, USA), according to manufacturer's instructions.

### 2.15 Surface plasmon resonance (SPR) biosensor analysis

The affinity determination of berberine binding to LDHA was measured using a Biacore T200 biosensor (GE Life, Chicago, IL, USA). The his‐tag LDHA human recombinant protein (Cat. No. 71081, BPS Bioscience, San Diego, CA, USA) was covalently immobilized on to the series S sensor chip CM5 (BR100530, GE Life, Chicago, IL, USA) with the amine coupling kit (BR100050, GE Life) in 20 mM MES buffer (pH 6.0) followed by deactivation of the residual amines by 1 M ethanolamine. Serial dilution of berberine (1.5625‐25 μΜ) was prepared by dilution in running buffer (20 mM Na phosphate, 150 mM NaCl, 0.005% v/v surfactant P20, 5% DMSO, pH7.4) at a flow rate of 30 μL/ min. The immobilized ligand was regenerated by injecting 10 mM Glycine‐HCl buffer (pH 2.2) for a time period allowing the response to return to baseline. The equilibrium binding data were analyzed for binding affinity of berberine to LDHA to generate the apparent dissociation constants (*K_d_*).

### 2.16 In‐vivo studies

Four‐ to six‐week‐old female C57BL/6N and BALB/cAnN‐nu(nude) mice sourced from Charles River Lab (Wilmington, M.A., USA) and inbred by our institutional laboratory animal unit were obtained for subsequent experiments. The animal protocols for the experiments were approved by the Committee on the Use of Live Animals in Teaching and Research of the University of Hong Kong (CULATR; No. 4577‐16) and the Ethics Committee of the Department of Laboratory Animal Science, Fudan University, Shanghai, China (Ref. No. 20160673A040).

#### 2.16.1 Orthotopic mouse model of pancreatic cancer

The orthotopic pancreatic tumor model was established according to our previous study with minor modifications.^19^ Briefly, 1 × 10^7^ pancreatic cancer cells were suspended in 0.1 mL of ice‐cold serum‐free medium and mixed with Matrigel matrix on ice (1:1, v/v) (No. 354234; Corning). Mice were anesthetized with 100 mg/kg ketamine and 10 mg/kg xylazine, following laparotomy of the left flank of abdominal skin and exposure of the spleen and the pancreatic body. Twenty microliter cell‐Matrigel mixtures was slowly injected into the pancreatic head for inoculation. Then, the pancreas and the spleen were placed back into the abdomen, and the muscle and skin were closed layer‐by‐layer with sutures. The tumor were allowed to grow for 7 days, and mice were randomized into berberine treatment 5 mg/kg group (5 mg/kg/2 days, oral gavage for 4 weeks, *n* = 10), berberine treatment 10 mg/kg group (10 mg/kg/2 days, oral gavage for 4 weeks, *n* = 10), and another group (*n *= 10) was subjected with the same volume of phosphate‐buffered saline (PBS; Gibco) as control. Bodyweight is monitored weekly and the mice were sacrificed after 4 weeks. Blood samples were withdrawn by cardiac puncture when the experimental animals were under deep anesthesia with ketamine/xylazine mixture. By the end of the 4‐week treatment, tumor was then removed and measured.

#### 2.16.2 Liver metastasis mouse model of pancreatic cancer

Establishment of the liver metastasis pancreatic tumor model as previously reported with slight modifications.^20^ In brief, 1 × 10^6^ pancreatic cancer cells were suspended in 0.1 mL of ice‐cold PBS (Gibco). Laparotomy technique was performed as aforementioned and 0.2 mL of cell suspension was slowly injected into the spleen. After inoculation, the tumors were allowed to grow for 28 days and the mice were sacrificed by injecting 200 mg/kg pentobarbital intraperitoneally and the livers were dissected out. The surface metastases on the livers were counted after dissection of the livers into individual lobes.

### 2.17 Histology, immunohistochemistry, and immunofluorescent analyses

Paraffin‐embedded tissues were sectioned at 4 μm thickness and mounted on slides. The slides were deparaffinized with xylene for twice, and rehydrated with 100%, 95%, 70%, and 50% ethanol, respectively for two changes. For visualization of histology, hematoxylin and eosin (H&E) staining was performed. Briefly, the rehydrated slides were submerged and incubated in Mayer's hematoxylin for 5 minutes. Afterward, slides were incubated in 1% acid alcohol for one second, rinsed in tap water and stained in 0.25% eosin Y solution (Sigma‐Aldrich) for 30 seconds. After staining, slides were then dehydrated with increasing concentrations of alcohol (50%‐100%). The stained slides were mounted in Canada balsam (Sigma‐Aldrich) and visualized under a microscope (Leica Microsystems Digital Imaging, Wetzlar, Germany).

For immunohistochemistry (IHC) staining, slides were incubated with respective anti‐ki‐67(1:200, ab15580, Abcam) and anti‐LDHA (1: 200, ab101562, Abcam) in a humidified chamber at 4°C overnight followed by incubation with respective diluted secondary antibody at room temperature for 1 hour. The slides were then stained with DAB substrate kit (vector laboratories, Burlingame, CA, USA) according to manufacturer's instructions and then counter stained with hematoxylin for 1 minute. After staining, slides were dehydrated, mounted, and visualized under the microscope.

For immunofluorescent staining, slides were incubated with APC conjugated CD3 antibody (1:200, 17‐0032, eBioscience, San Diego, CA, USA), Alexa Fluor 488‐conjugated CD4 antibody (1:200, 53‐0041, eBioscience) and Alexa Fluor 700‐conjugated CD8a antibody (1:200, 56‐0081, eBioscience) in a humidified chamber at 4°C overnight followed by counter staining with DAPI (Cat. D1306, Invitrogen) at room temperature for 3 minutes. Stained slides were mounted with fluorescent mounting medium (Dako, Denmark) and visualized under a confocal microscope (Carl Zeiss LSM780, Germany).

### 2.18 Statistical analysis

The matching and significance of patients clinical‐pathological data were assessed using Student's *t*‐test (two‐tailed), chi‐square test, Mann‐Whitney *U* Test, Pearson's correlation coefficient, receiver operating characteristic (ROC) curve, multivariate Cox proportional hazards regression, and Kaplan‐Meier (K‐M) analyses, where appropriate, by SPSS software version 20.0 (IBM Corporation, Chicago, IL, USA). For the in vitro and in vivo data, the significance was determined using the Student's *t*‐test (two‐tailed) or one‐way analysis of variance (one‐way ANOVA) using GraphPad Prism 7.0 (GraphPad Software, San Diego, CA, USA), where appropriate. A *P* value of less than 0.05 was considered statistically significant. All the experiments were performed independently in triplicate unless otherwise specified.

## 3 RESULTS

### 3.1 The overexpression of LDHA in pancreatic cancer associated with poor prognosis

Glycolysis is a hallmark of malignancy transformation in solid tumor, and LDH is the key enzyme involved in glycolysis. To determine the clinically relevant role of lactate dehydrogenase in PAAD patients, we retrieved transcriptomic expression profiles of PAAD tumor and adjacent nontumoral normal tissues from the GEO dataset (GDS4103 and GDS4336) and the Oncomine database. The mRNA expression of LDHA was significantly upregulated in tumor tissues compared to the adjacent nontumoral normal tissue (Figure S1A, Table S2). On the other hand, the mRNA expression of LDHB showed reduced expression in cancerous tissues compared to the adjacent noncancerous normal tissues. No significant difference in LDHC expression between cancerous tissue and nontumoral tissue was found. Further analysis on the clinical correlation of LDH with OS and DFS of PAAD patients showed that patients with high expression of LDHA mRNA expressions are associated with poorer OS and DFS (HR = 2.2, *P* = 0.00028; HR = 2.1, *P* = 0.00087, respectively) (Figure S1B). These data demonstrate that LDHA is overexpressed in pancreatic cancer compared to its normal adjacent tissue, and LDHA overexpression results in poor clinical outcomes in pancreatic adenocarcinoma patients.

### 3.2 Serum LDH isoenzyme 5 expressions are associated with an unfavorable prognosis and clinical progression

**FIGURE 1:**
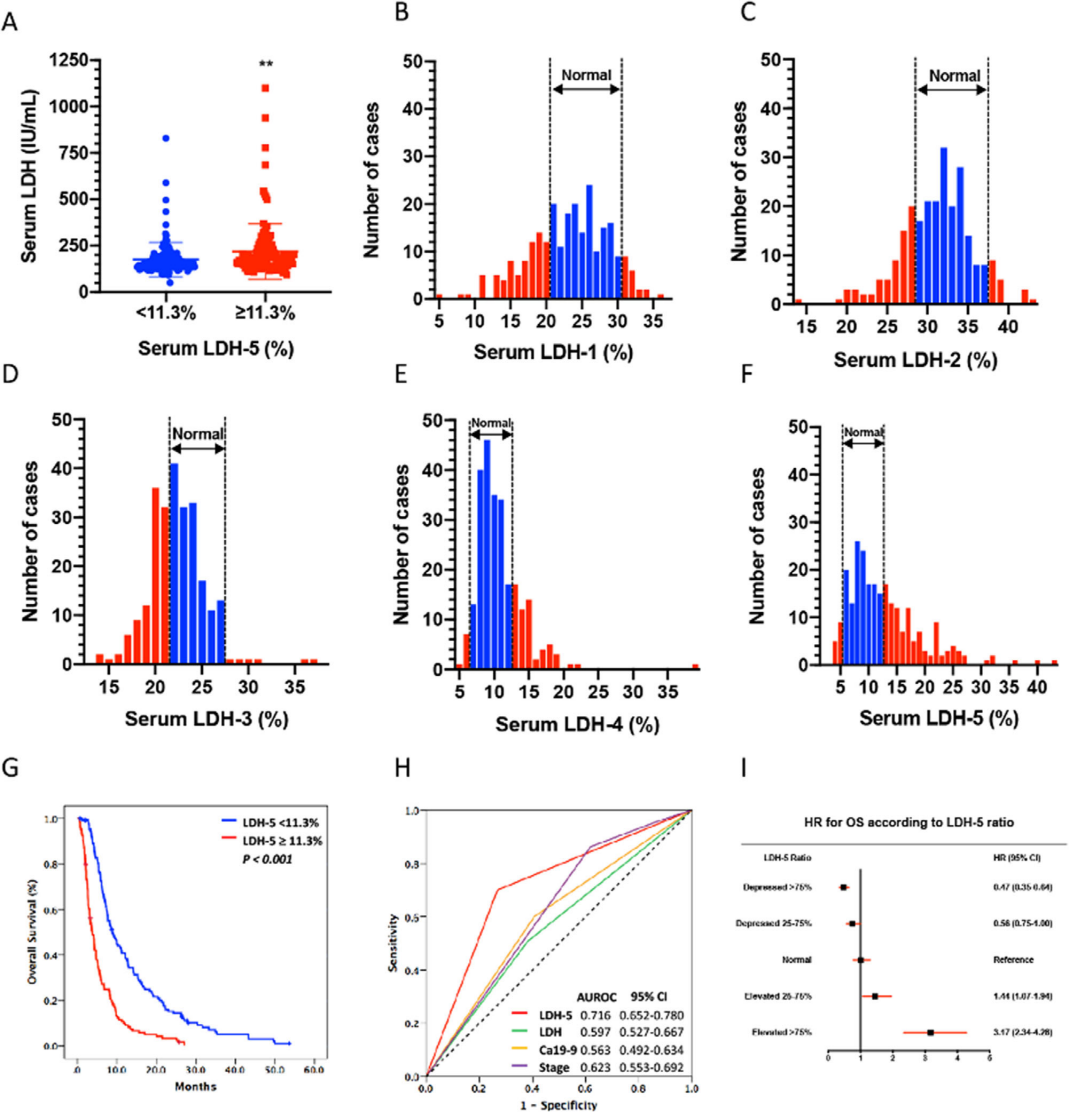
Elevated serum LDHA‐transcribed isoenzymes‐5 (LDH‐5) was associated with poorer patients’ clinical outcomes. (A) The serum LDH is significantly higher in those who had serum LDH‐5 ≥ 11.3% than in those who had serum LDH‐5 < 11.3% (*n* = 253, **P < 0.01). (B) The distribution of serum LDH isoenzymes‐1 of the included patients. LDH‐1 accounting for 20.0%‐31.0% of all isoenzymes are regarded as normal (*n* = 253). (C) The distribution of serum LDH isoenzymes‐2 of the included patients. LDH‐2 accounting for 28.8%‐37.0% of all isoenzymes are regarded as normal (*n* = 253). (D) The distribution of serum LDH isoenzymes‐3 of the included patients. LDH‐3 accounting for 21.5%‐27.6% of all isoenzymes are regarded as normal (*n* = 253). (E) The distribution of serum LDH isoenzymes‐4 of the included patients. LDH‐4 accounting for 6.3%‐12.4% of all isoenzymes are regarded as normal (*n* = 253). (F) The distribution of serum LDH isoenzymes‐5 of the included patients. LDH‐5 accounting for 5.4%‐13.2% of all isoenzymes are regarded as normal (*n* = 253). (G) Kaplan‐Meier(KM) plots illustrate overall survival (OS) for in patients with pancreatic cancer who had serum LDH < 161IU/L and LDH ≥ 161IU/L, and serum LDH‐5 < 11.3% and LDH‐5 ≥11.3%. (H) Area under the receiver operating characteristic (AUROC) curves of the sensitivity and specificity of survival prediction by serum LDH isoenzyme 5 (LDH‐5), serum LDH (LDH), cancer antigen 19‐9(Ca19‐9) and clinical stage. (I) A prognostic value of LDH‐5 ratio with hazard ratio (HR) and 95% confidence interval (95% CI) for overall survival (OS). “Normal” (defined as LDH‐5 from –25% to +25%) was used as the reference category

To further investigate the relationship between serum LDH and prognosis of pancreatic cancer patients, we retrospectively reviewed the clinical characteristics of 253 patients (Table 1). The median serum LDH level among the patients is 161.0 IU/L (*SD*: 124.79; range 50‐1099.0). KM survival analysis revealed a median OS of 4.733 ± 0.568 months (95% CI: [3.620‐5.847]) for those who had serum LDH ≥ 161.0 IU/L and 7.467 ± 0.623 months for those who had serum LDH < 161.0 IU/L (log‐rank 5.239; *P* = 0.022). The median serum LDH‐5 is 11.3% (*SD*: 6.504; range 3.55‐43.00). The serum LDH is significantly higher in those who had serum LDH‐5 ≥ 11.3% than in those who had serum LDH‐5 < 11.3% (*P* < 0.01)(Figure 1A). The distribution of serum LDH isoenzymes‐1 to ‐5 of the included patients is shown in Figure 1B‐F. The median OS for patients was 3.800 ± 0.406 months (95% CI: [3.004‐4.596]) for those who had serum LDH‐5 ≥ 11.3% and 8.80 ± 0.959 (95% CI: [6.920‐10.680]) months for those who had serum LDH‐5 < 11.3% (log‐rank, 56.494; *P* < 0.001) (Figure 1G). Clinical characteristics of patients based on LDH‐5 level are shown in Table 2.

**TABLE 1:** Clinical characteristics of patients

Characteristic	Total (*n *= 253)	Low LDH‐5 < 11.3%*n *= 129 (51.0%)	High LDH‐5≥ 11.3%*n *= 124 (49.0%)	*P*
Age (years)				
Mean (*SD*)	60.95 (10.08)	61.02 (9.6)	61.85 (9.56)	0.500^a^
< 60	106 (41.90)	54	52	0.990^b^
≥60	147 (58.10)	75	72	
Gender, *n* (%)				0.690^b^
Male	156 (61.66)	78	78	
Female	97 (38.34)	51	46	
BMI	22.07 (2.81)	22.12 (2.88)	22.03 (2.75)	0.804^a^
Clinical stage, *n* (%)				**<0.003** ^b^
IIb	23 (9.09)	17	6	
III	42 (16.60)	28	14	
IV	188 (74.31)	84	104	
Tumor location, *n* (%)				
Head and neck	100 (39.53)	51	49	0.998^b^
Body and tail	153 (60.47)	78	75	
Tumor diameter (mm)				
Mean (*SD*)	43.33 (15.98)	43.76 (14.43)	49.10 (17.13)	**0.008** ^c^
Ca19‐9 (IU/mL)				
Mean (*SD*)	549.1 (435.7)	540.1 (424.2)	619.6 (445.4)	0.165^c^
<1000	140 (55.34)	80	60	**0.029** ^b^
≥1000	113 (44.66)	49	64	
LDH (IU/L)				
Mean (*SD*)	196.4 (124.8)	175.28 (91.73)	218.36 (148.98)	**0.001** ^c^
AST/ALT				
Mean (*SD*)	1.15 (0.52)	1.10 (0.41)	1.21 (0.60)	0.429^c^
Overall survival (months)				
Median (*SD*)	6.10 (0.45)	8.80 (0.96)	3.80 (0.41)	**<0.0001** ^d^
Liver metastases, *n* (%)				
Presence	162 (64.03)	67	95	**<0.001** ^b^
Absence	91 (35.97)	62	29	
Lung metastases, *n* (%)				
Presence	45 (17.79)	20	25	0.333^b^
Absence	208 (82.21)	109	99	
Bone metastases, *n* (%)				
Presence	22 (8.70)	7	15	0.060^b^
Absence	231 (91.30)	122	109	
Retroperitoneal lymph node metastases, *n* (%)				
Presence	164 (64.82)	84	80	0.920^b^
Absence	89 (35.18)	45	44	
Received gemcitabine‐based chemotherapy, *n* (%)				
Yes	199 (78.66)	108	91	**0.045** ^b^
No	54 (21.34)	21	33	
Received S‐1 chemotherapy, *n* (%)				**0.013** ^b^
Yes	118 (46.64)	70	48	
No	135 (53.36)	59	76	
Received ablation therapy^e^, *n* (%)				**0.001** ^b^
Yes	133 (52.57)	81	52	
No	120 (47.43)	48	72	
CM treatment^f^, *n* (%)				
Yes	136 (53.75)	74	62	0.240^b^
No	117 (46.25)	55	62	

*Bolded text indicates a statistically significant difference with a *P* value less than 0.05.

Abbreviations: LDH, lactate dehydrogenase; LDH‐5, lactate dehydrogenase 5; BMI, body mass index; Ca 19‐9, cancer antigen 19‐9; SD, standard deviation; AST, aspartate transaminase; ALT, alanine transaminase.

a This *P* value was determined using Student's *t*‐test.

b This *P* value was determined using Pearson's chi‐square test.

b This *P* value was determined using the Mann‐Whitney *U* test.

d This *P* value was determined using log rank (Mentel‐Cox).

e Ablation therapy included radiofrequency ablation or high‐intensity focused ultrasound ablation of the liver metastatic lesion, retroperitoneal lymph node metastatic lesion.

f Chinese Medicine (CM) treatment included granules or herbal decoction.

**TABLE 2:** Univariate and multivariate analysis of the LDH‐5 for predicting overall survival

						95% CI for Exp (*B*)	
Variable	*B*	*SE*	Wald	Sig.	Exp(*B*)	Lower	Upper
**Univariate analysis**							
Age (< 60 vs ≥60)	0.001	0.007	0.008	0.929	1.001	0.987	1.014
Gender (male vs female)	–0.256	0.145	3.117	0.077	0.774	0.583	1.029
BMI	0.013	0.025	0.262	0.609	1.013	0.965	1.063
Clinical stage	0.301	0.110	7.478	**0.006**	1.351	1.089	1.677
Tumor location (head/neck vs body/tail)	0.1230	0.144	0.814	0.367	1.138	0.859	1.508
LDH‐5 (< 11.3% vs ≥11.3%)	1.004	0.146	47.501	**<0.001**	2.730	2.052	3.633
Ca19‐9	0.000	0.000	5.107	**0.024**	1.000	1.000	1.001
AST/ALT	0.133	0.144	0.843	0.358	1.142	0.860	1.515
Received gemcitabine‐based chemotherapy (yes vs no)	−0.355	0.181	3.856	**0.050**	0.701	0.492	0.999
Received oral chemotherapy (yes vs no)	−0.345	0.146	5.619	**0.018**	0.708	0.532	0.942
CM treatment (yes vs no)	−0.339	0.136	6.215	**0.013**	0.713	0.546	0.930
**Multivariate analysis**							
Clinical stage	0.279	0.106	6.982	**0.008**	1.322	1.075	1.626
Ca19‐9	0.000	0.000	5.179	**0.023**	1.000	1.000	1.001
LDH‐5 (< 11.3% vs ≥11.3%)	0.998	0.141	50.183	**<0.001**	2.712	2.058	3.573
Received gemcitabine‐based chemotherapy (yes vs no)	−0.384	0.172	4.975	**0.026**	0.681	0.486	0.955
Received oral chemotherapy (yes vs no)	−0.331	0.140	5.590	**0.18**	0.718	0.546	0.945
CM treatment (yes vs no)	−0.297	0.133	5.016	**0.025**	0.743	0.573	0.964

*Bolded text indicates a statistically significant difference with a *P* value less than 0.05.

Abbreviations: LDH‐5, lactate dehydrogenase 5; BMI, body mass index; Ca 19‐9, cancer antigen 19‐9; SD, standard deviation; AST, aspartate transaminase; ALT, alanine transaminase; CM, Chinese Medicine; CI, confidence interval.

Note: Predicting model for poor prognosis in patients with pancreatic cancer *Y* = *h*(*t*)/*h*(*t*_0_) = exp(6.982 × Clinical stage + 5.179 × Ca19‐9 + 50.183 × LDH‐5 – 4.975 × Received gemcitabine‐based chemotherapy – 5.590 × Received Oral Chemotherapy – 5.016 × CM treatment). Indicating that patients with advanced clinical stage, elevated serum Ca19‐9, and serum LDH isoenzyme 5 (LDH‐5) over 11.3%, are at a 6.982‐fold, 5.179‐fold, and 50.183‐fold, respectively, risk of poor prognosis. Patients received gemcitabine‐based chemotherapy, oral chemotherapy, and CM treatment are a 4.975‐fold, 5.590‐fold, 5.016‐fold rick of good prognosis.

In order to determine the clinical usefulness of serum LDH‐5 as a biomarker, the Cox regression proportional hazard analyses were employed to assess the relative risks. As shown in Table 2, the univariate Cox regression analyses showed that a high level of serum LDH‐5 was associated with an increased risk of death in pancreatic cancer patients with an odds ratio (OR) of 2.730 (95% CI: [2.052‐3.633]) (*P* < 0.001). The relative risk increased by over 47.5‐fold in patients with high LDH‐5 levels compared to those with low LDH‐5 levels (Table 2). As anticipated, advanced clinical stage (*P* = 0.006) and elevated Ca19‐9 level (*P* = 0.024) were also significant poor prognostic factors. Further multivariate analysis of the relative risks indicative of LDH‐5′s prognostic role in pancreatic cancer showed that patients with higher tumor stage, elevated serum Ca19‐9, and serum LDH‐5 over 11.3%, are at a 6.982‐fold, 5.179‐fold, and 50.183‐fold, respectively, risk of poor prognosis. The ROC analysis confirmed that LDH‐5 had a better prognostic value than other clinical parameters (Figure 1H). The prognostic value of LDH‐5 was calculated based on the median level of all included patients and subsequently grouped into five groups (>7 5% depressed, 25%‐75% depressed, normal [from <25% depressed to <25% elevated], 25%‐75% elevated, and > 75% elevated) with the calculation of the hazard ratio (HR) per group. We observed that, compared to normal (from < 25% depressed to < 25% elevated), elevated for 25%‐75%, and > 75% in LDH‐5 was associated with poor OS (HR: 1.44, 95% CI: [1.07‐1.94] and HR: 3.17, 95% CI: [2.34‐4.28], respectively) (Figure 1I).

To further identify if serum LDH and serum LDH‐5 level affect pancreatic cancer patients’ progression, we sought to determine the correlation of serum LDH with time to pancreas‐to‐liver metastasis. However, as most of the present study patients presented with synchronous liver metastasis upon diagnosis, and only 28 patients developed liver metastasis after diagnosis, we failed to identify a statistical. We, therefore, analyzed the diagnostic likelihood ratio (DLR) of serum LDH with liver metastasis. A serum LDH level of 195.5 IU/L correlated with 100% sensitivity for detecting liver metastasis with an AUC of 0.662 (95% CI: [0.595‐0.729]), with a positive DLR of 3.932. As for serum LDH‐5, a level of 9.25% correlated with 100% sensitivity for detecting liver metastasis with an AUC of 0.669 (95% CI: [0.630‐0.768]), with a positive DLR of 1.928. Furthermore, our analysis showed that LDH‐5 is positively correlated with tumor size (*P = *0.029). Together, these results suggest that LDHA overexpression is associated with clinical disease progressions and malignant behaviors of pancreatic cancer.

### 3.3 LDHA overexpression enhances cell proliferation, migration, and invasion of pancreatic cancer cells

**FIGURE 2:**
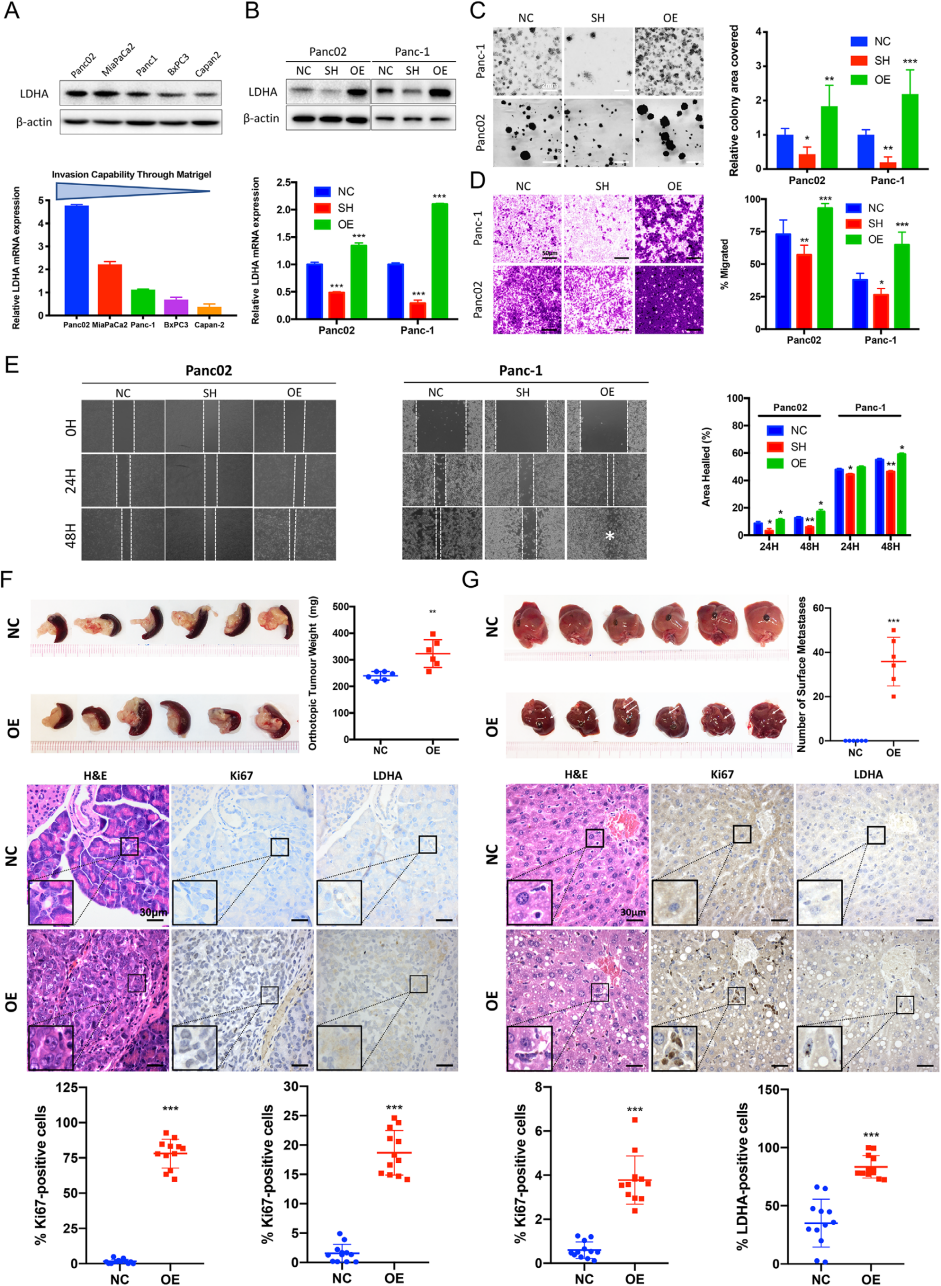
Exogenous overexpress LDHA enhances proliferation, migration, and invasion in vitro and promotes tumorigenesis and liver metastasis in vivo. (A) The protein expression of LDHA of various pancreatic cancer cell lines was confirmed by immunoblotting. *Beta‐actin* was used as loading control. The mRNA expression of LDHA and invasion capability of various pancreatic cancer cell lines. Invasion capability through Matrigel and ordered from strongest to lowest, from Panc02 cells to Capan‐2 cells (*n *= 3 per group). (B) The overexpression and knockdown of LDHA‐transfected Panc02 and Panc‐1 cells was confirmed by immunoblotting and RT‐PCR. Beta‐actin was used as loading control (*n* = 3 per group). (C) LDHA overexpression (OE) promoted the colony formation capabilities in Panc02 and Panc‐1 cells (*n* = 6 per group, **P < 0.01, ***P < 0.001) compared with controlled cells (NC). LDHA knockdown (SH) suppressed colony formation in Panc‐1 and Panc02 cells (*n* = 6 per group, *P < 0.05, **P < 0.01) compared to controlled cells (NC). (D) The invasive properties were analyzed by Matrigel‐coated Boyden chamber assay and scored under a light microscope (200×). LDHA knockdown (SH) suppressed the invasive properties in Panc02 cells (**P < 0.01) and Panc‐1 cells (*P < 0.05) compared to the respective controlled cells (NC) (*n* = 6 per group). Overexpression of LDHA (OE) promoted cell invasion in both Panc02 and Panc‐1 cells (*n* = 6 per group, ***P < 0.01) compared to the respective controlled cells (NC). (E) A wound was produced and monitored at 0, 24, and 48 h as the cells moved and filled the damaged area (200×). The data were plotted as the percentage area healed (*n* = 3 per group, *P < 0.05, **P < 0.01). (F) The tumor size and average weight of primary xenografts of orthotopic implantation model (*n* = 6 per group, **P < 0.01). H&E staining confirmed the tumorigenesis of LDHA‐OE cells in orthotopic model (400×). The Ki‐67 and LDHA expression in the histological sections was detected by immunohistochemical (IHC) staining (*n* = 12 per group, ***P < 0.001, 400×). (G) Macroscopic liver metastasis lesion was visible on the surface of the liver tissue (white arrowed) in LDHA‐OE group and the number of liver metastatic lesions in mice (*n *= 6 per group) was analyzed by counting the macroscopic lesion from each hepatic lobe (***P < 0.001). H&E staining identified adenocarcinoma in the liver section of the LDHA‐OE group, while no liver metastasis lesion was identified with the control group (400×). The Ki‐67 and LDHA expression in the histological sections was detected by immunohistochemical (IHC) staining (*n* = 12 per group, 400×). NC: controlled cells; SH: LDHA knockdown cells; OE: LDHA overexpressed cells

Cell proliferation, migration, and invasion are requisites for cancer progression and metastasis. As our clinical analysis showed a positive correlation between serum LDH‐5 level and a larger tumor size, the presence of liver metastasis, and a more advanced disease stage in pancreatic cancer patients, we hypothesized that its encoding gene, LDHA, may regulate pancreatic cancer progression. To determine the effect of LDHA expression on pancreatic cancer, we first analyzed the mRNA expression and invasion capability of various pancreatic cancer cell lines.^21^ It was observed that the LDHA mRNA expression was lower in the pancreatic cancer cell line with reduced invasion capability. Consistently, we found that the protein expression of LDHA is highly detected in the highly metastatic cell line Panc02, medially expressed in Panc‐1 cell line, and lowly expressed in the weakly metastatic Capan‐2 cells (Figure 2A). In order to address the above‐mentioned hypothesis, LDHA CRISPR/Cas9 activation and knockdown plasmids, and LDHA‐OE and shRNA lentivirus were stably transfected into Panc‐1 and Panc02 cells, respectively, and verified at both mRNA and protein level (Figure 2B). LDHA overexpression promoted colony formation in the pancreatic cancer cells, suggesting a promoted proliferation (Figure 2C). Wound healing assays and Boyden chamber assays were performed to determine the potential of LDHA to induce cell migration and invasion, respectively. The results showed that the overexpression of LDHA increased cell invasion (Figure 2D) and migration (Figure 2E) compared to the control. Meanwhile, the knockdown of LDHA impeded pancreatic cancer cell functions (Figure 2C‐E).

To further assess the effects of LDHA on pancreatic cancer tumorigenesis and metastasis in vivo, LDHA‐NC, LDHA‐OE, and LDHA‐SH Panc‐1 cells were injected into the pancreas and spleen of BALB/cAnN‐nu (nude) mice to generate orthotopic implantation model and liver metastasis model. Macroscopic xenografts were observed in the pancreatic tissue and liver of nude mice injected with LDHA‐OE Panc‐1 cells after 4 weeks, with significantly higher tumor weight than the wild‐type control (*P* < 0.05). As assessed by H&E staining, the orthotopic xenografts from LDHA‐OE Panc‐1 cells invaded the pancreatic duct and adjacent tissue (Figure 2F). In the liver metastasis model, macroscopic liver metastasis lesions were visible on the surface of the liver tissue in the LDHA‐OE group. Subsequent H&E staining identified adenocarcinoma in the liver section of the LDHA‐OE group, while no liver metastasis lesion was identified with the control group (Figures 2G and S2). On the other hand, no orthotopic nor liver metastasis lesions were observed on the LDHA‐SH Panc‐1 models (Figure S3). An increase in proliferation in the LDHA‐OE group was found in both tumor and liver tissues compared to that of the vehicle control, as observed by detecting the expression level of Ki67, a marker of cell proliferation (Figure 2F‐G). Similarly, we also observed an increase in LDHA expression in both tumor and liver tissues compared to that of the control (CTL) group mice (Figure 2F‐G). These results, therefore, suggest that LDHA overexpression promoted the proliferation and metastasis of pancreatic cancer.

### 3.4 LDHA‐induced L‐lactate production is responsible for the accelerated proliferation and invasion of PAAD cells

**FIGURE 3:**
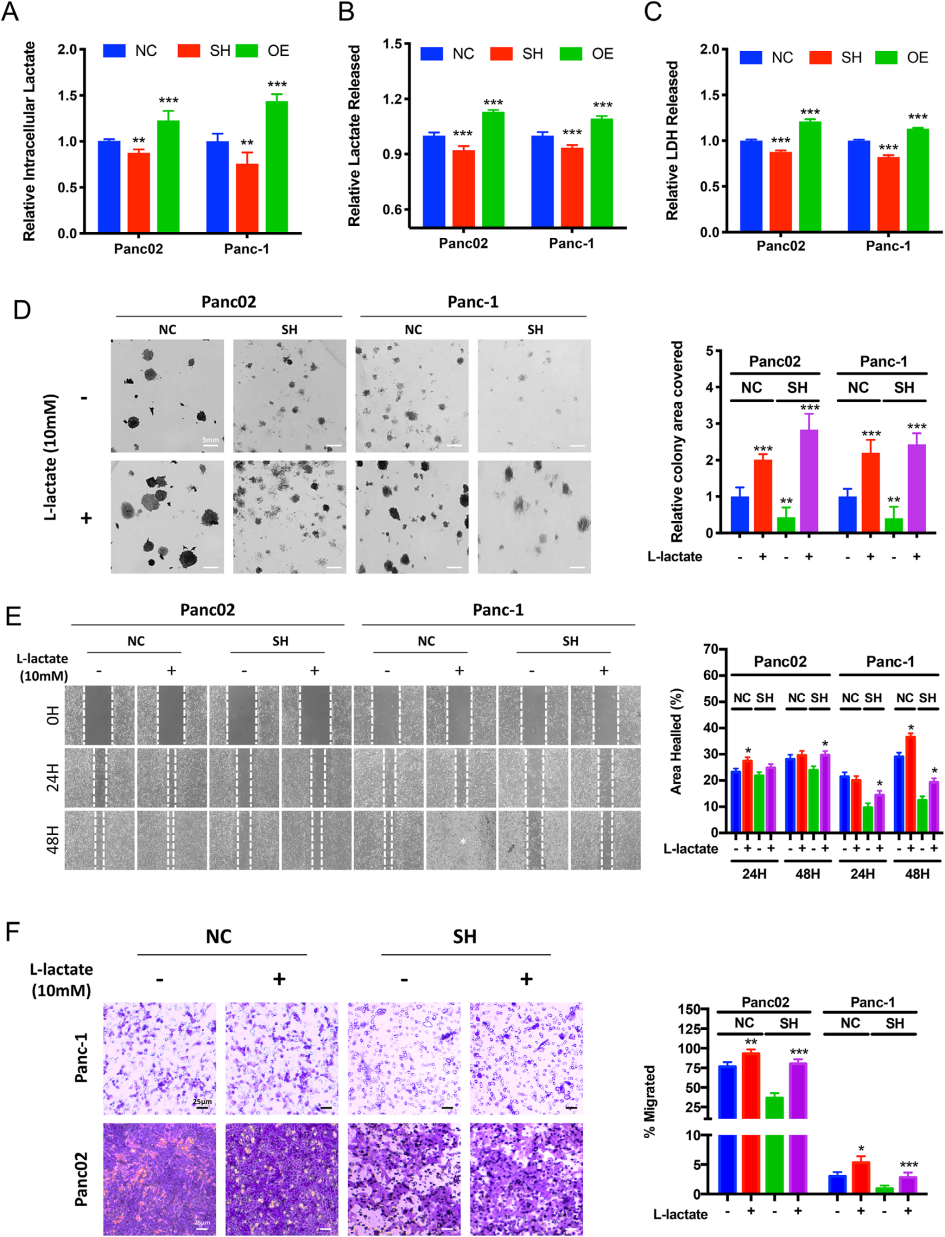
Promoting LDHA enzymatic activity enhances proliferation, migration and invasion ability of LDHA knockdown cells. (A) Intracellular lactate assay was performed with cells with exogenous LDHA expression. The graph represents the relative intensity of lactate detected intracellularly in LDHA overexpressed (OE) and knockdown (SH) cells compared with that of the controlled (NC) cells (*n* = 6 per group, **P < 0.01, ***P < 0.001). (B) Extracellular lactate assay was performed with cells with exogenous LDHA expression. The graph represents the relative intensity of lactate detected in the culture supernatant of LDHA overexpressed (OE) and knockdown (SH) cells compared with that of the controlled (NC) cells normalized per cell numbers (*n *= 6 per group, ***P < 0.001). (C) In vitro LDH release assay was performed to assess LDH enzymatic activity among cells with differential LDHA expression. The graph represents the relative intensity of LDH released detected in the culture supernatant (*n *= 6 per group, ***P < 0.001). (D) Colony formation assay was performed with controlled cells (NC) and LDHA knockdown (SH) cells supplement with 10 mM L‐lactate. The graph represents the percentage of area covered by colonies after 10 days of incubation (*n* = 6 per group, **P < 0.01, ***P < 0.001). (E) Scratch wound healing assay was performed controlled (NC) cells and LDHA knockdown (SH) cells supplement with 10 mM L‐lactate. The graph represents the wound confluence percentage after 24 and 48 hours of incubation (*n* = 3 per group, *P < 0.05). (F) Boyden chamber invasion assay was performed for 24 hours with controlled (NC) cells and LDHA knockdown (SH) cells with or without 10 mM L‐lactate. The graph represents the percentage of area covered by migrated cells after incubation (*n* = 6 per group, *P < 0.05, **P < 0.01, ***P < 0.001). NC: controlled cells; SH: LDHA knockdown cells; OE: LDHA overexpressed cells

As aforementioned, LDHA favors the catalyzation of pyruvate to L‐lactate. To further investigate the association of LDHA expression with L‐lactate production in pancreatic cancer, we measured the intracellular and extracellular L‐lactate concentration by lactate assays and performed in vitro LDH release assay to assess the LDH enzymatic activity in PAAD cells. The intracellular and extracellular lactate assay both showed a decreased extracellular L‐lactate release in LDHA knockdown cells(*P* < 0.05), while a marked increase in both intracellular and extracellular L‐lactate concentration in the LDHA overexpressed cells (*P* < 0.01) as compared to control cells (Figure 3A and B). Consistently, a similar trend was observed in the in vitro LDH release enzymatic activity assay (Figure 3C), suggesting a compromised LDH enzymatic activity in the LDH‐depleted cells, while enzymatic activity was promoted in the overexpressed cells.

To further explore whether LDHA‐induced L‐lactate production is responsible for the accelerated proliferation and invasion of PAAD cells, we supplemented our selected noncytotoxic dose of L‐lactate (Figure S4), 10 mM, to Panc02 and Panc‐1 cells with depleted LDHA. We observed a restored clonogenic capabilities in both knockdown cell lines (*P* < 0.05) (Figure 3D). Consistently, in LDHA overexpressed cell, the addition of 7ACC2, a MCT1 inhibitor suppressed the clonogenic capabilities of PAAD cells (Figure S5A). Next, to determine the effects of L‐lactate on the migration potential of LDHA knockdown cells, we performed scratch wound healing assay. After a 24‐hour incubation, compared with LDHA knockdown cells, L‐lactate supplementation led to a restoration of migration potential (Figure 3E) (*P* < 0.05). Furthermore, Matrigel‐coated Boyden chamber invasion assays showed L‐lactate treatment promoted the invasive potential of nontransfected control cells, and markedly restored the invasion inhibition by LDHA knockdown (Figure 3F) (*P* < 0.001). Similarly, in LDHA‐overexpressed cell, 7ACC2 suppressed in invasive capability of PAAD cells (Figure S5B). These results collectively demonstrated that LDHA knockdown suppressed the proliferative, migratory, and invasive capability of PAAD cells, whereas restoration of its enzymatic activity reversed these functions, suggesting that LDHA‐induced L‐lactate production plays a critical role in PAAD progression.

### 3.5 LDHA‐induced L‐lactate‐regulated AMPK‐mTOR in PAAD cells

**FIGURE 4:**
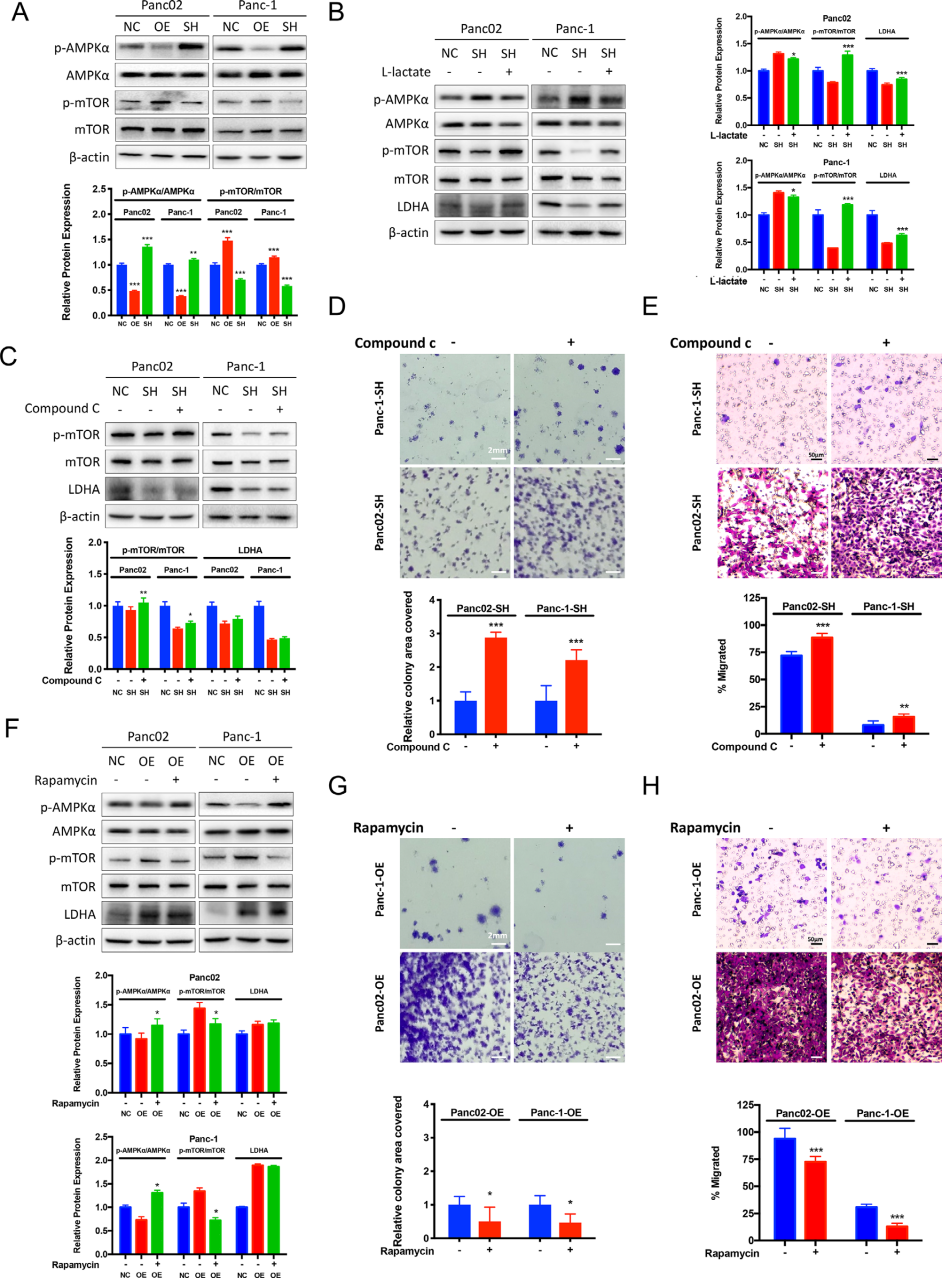
L‐lactate restores AMPK activation by LDHA knockdown and LDHA is an upstream of the AMPK‐mTOR pathway. (A) The expression of the indicated proteins was analyzed by immunoblotting. The graph represents the relative protein expression ratio of pAMPKa/AMPKa and pmTOR/mTOR normalized to *beta‐actin* compared to the controlled (NC) cells (*n* = 3 per group, **P < 0.01, ***P < 0.001). (B) LDHA knockdown (SH) cells were incubated with 10 mM L‐lactate for 24 hours. The graph represents the relative protein expression ration of pAMPKa/AMPKa and pmTOR/mTOR normalized to *beta‐actin* compared to the nontreated knockdown (SH) cells (*n* = 3 per group, *P < 0.05, ***P < 0.001). (C) LDHA knockdown (SH) cells were incubated with 20 μM AMPK inhibitor (Compound C) for 24 hours. The graph represents the relative protein expression ration of pAMPKa/AMPKa and pmTOR/mTOR normalized to *beta‐actin* compared to the nontreated knockdown (SH) cells (*n* = 3 per group, *P < 0.05, **P < 0.01). (D) Colony formation assay was performed with nontreated LDHA knockdown (SH) cells and LDHA‐SH cells supplement with 20 μM Compound C. The graph represents the percentage of area covered by colonies after 10 days of incubation (*n* = 6 per group, ***P < 0.001). (E) Boyden chamber invasion assay was performed for 24 hours with LDHA knockdown (SH) cells with or without 20 μM Compound C. The graph represents the percentage of area covered by migrated cells after incubation (*n* = 6 per group, **P < 0.01, ***P < 0.001). (F) LDHA overexpressed cells were incubated with 10 nm Rapamycin for 24 hours. The graph represents the relative protein expression ratio of pAMPKa/AMPKa and pmTOR/mTOR normalized to *beta‐actin* compared to the nontreated overexpressed (OE) cells (*n* = 3 per group, *P < 0.05). (G) Colony formation assay was performed with LDHA overexpressed (OE) cells supplement with or without 10 nm Rapamycin. The graph represents the percentage of area covered by colonies after 10 days of incubation compared to the nontreated overexpressed (OE) cells (*n* = 6 per group, *P < 0.05). (H) Boyden chamber invasion assay was performed for 24 hours with LDHA overexpressed cells treated with or without 10 nm Rapamycin. The graph represents the percentage of area covered by migrated cells after incubation (*n* = 6 per group, ***P < 0.001). NC: controlled cells; SH: LDHA knockdown cells; OE: LDHA overexpressed cells

To further delineate the mechanism involved in LDHA‐mediated PAAD progression, we performed western‐blot analysis on LDHA‐overexpressed or ‐knockdown pancreatic cancer cells. Noting that LDHA plays a critical role in glycolytic metabolism, we further found that the metabolic regulator, the p‐AMPKa/AMPK ratio is decreased in LDHA‐OE cells, while increased in LDHA‐SH cells(Figure 4A) accompanied by a significant increase of the cellular AMP/ATP ratio in LDHA‐SH cells (Figure S6). The finding further suggested that LDHA‐induced L‐lactate‐regulated AMPK activation in PAAD cells. Activation of AMPK was reported to suppress the mTOR activity; therefore, we further investigated the correlation between the p‐mTOR/mTOR ratio with LDHA expression. Expectedly, we found that the p‐mTOR/mTOR ratio is increased in LDHA‐OE cells, while decreased in LDHA‐SH cells (Figure 4A). Observing the activation of AMPKa in LDHA‐SH cells, we, therefore, hypothesized that LDHA‐induced L‐lactate regulated the PAAD growth through the AMPK/mTOR pathway. To further test the hypothesis, we first examined the expression of AMPKa and mTOR in LDHA‐SH cells treated with L‐lactate. At protein levels, we observed a significant restoration of AMPK activation, mTOR suppression, and LDHA expression in LDHA‐SH cells upon supplementation of L‐lactate (Figure 4B). Collectively, these data revealed that in LDHA‐SH cells, the restoration of LDHA enzymatic activity further suppressed the AMPK/mTOR pathway, LDHA‐induced L‐lactate regulated the AMPK/mTOR signaling in PAAD cells.

To confirm the role of the AMPK/mTOR signaling in regulating LDHA‐induced cancer cell progression, we then pharmacologically inhibited AMPK expression with 20 μM Compound C, a selective and cell‐permeable AMPK inhibitor, in LDHA‐SH cells. Immunoblotting assay confirmed that AMPK inhibition restored the mTOR activity, but LDHA expression was minimally detected on LDHA‐SH cells (Figure 4C). Functionally, the addition of compound C in LDHA‐SH cells reversed the growth and invasion inhibition by LDHA blockade (Figure 4D and E). Consistently, blockade of mTOR activity by 10 nM of Rapamycin activates AMPKa and suppresses mTOR in LDHA‐OE cells (*P* < 0.05) (Figure 4F). On the other hand, Rapamycin showed no inhibition on LDHA protein expression in LDHA‐OE cells (Figure 4F). Functionally, the inhibition of mTOR on LDHA‐OE cell with Rapamycin suppressed the colonial formation capabilities (Figure 4G) and invasion (Figure 4H) of pancreatic cells induced by LDHA overexpression. Our results reveal that the AMPK/mTOR signaling may be the downstream signaling of LDHA‐mediated proliferation and metastasis in PAAD cells.

### 3.6 Berberine as an LDHA inhibitor plays critical role in suppressing LDHA/AMPK‐mTOR mediated cancer cell progression

**FIGURE 5:**
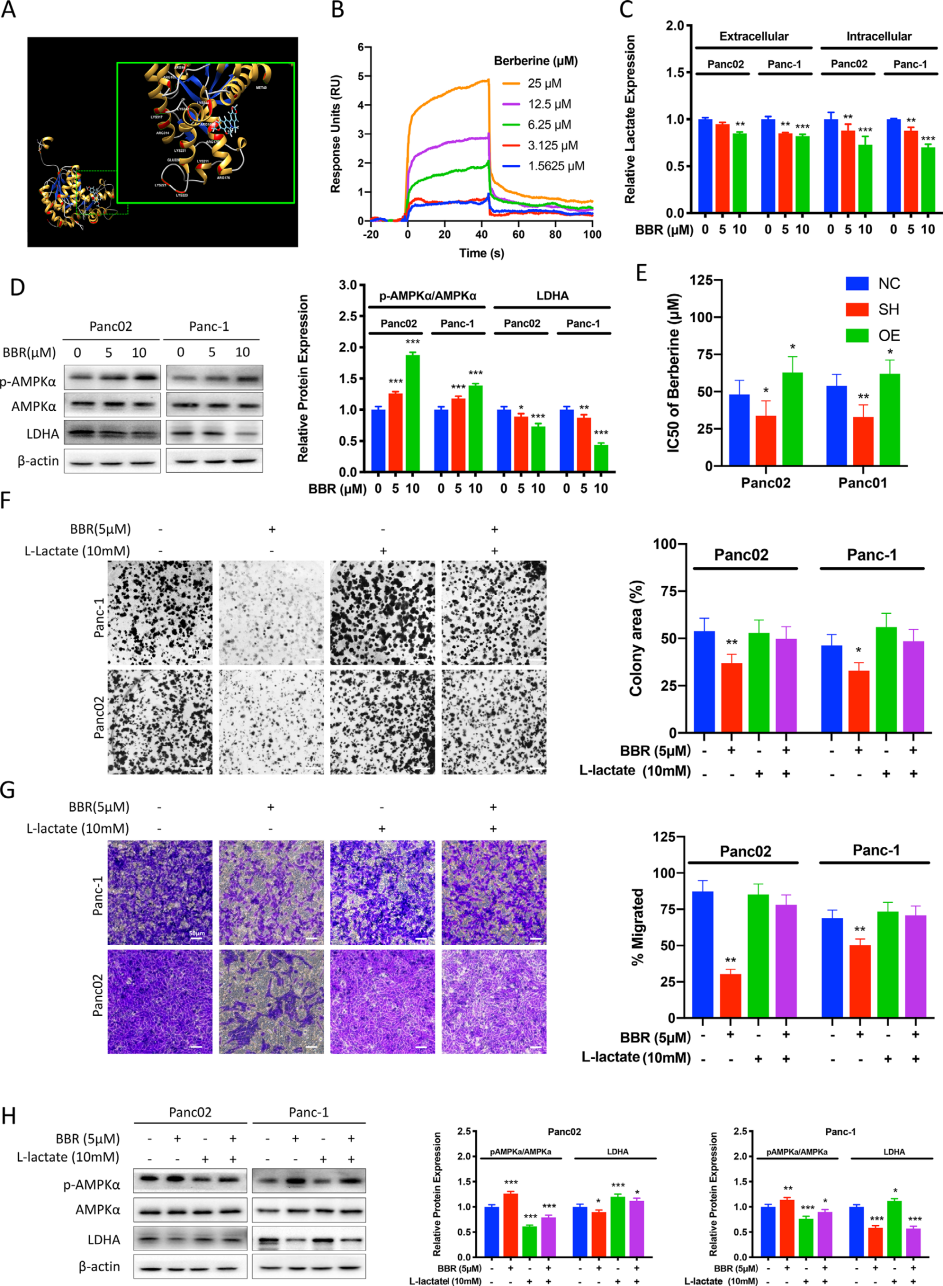
Berberine suppressed pancreatic cancer proliferation and invasion by LDHA/AMPK pathway. (A) Berberine may direct bind to LDHA, as predicted by molecular docking. (B) Surface plasmon resonance (SPR) biosensor analysis of berberine binding to LDHA. The sonograms for binding of berberine in serial concentration (1.5625, 3.125, 6.25, 12.5, 25 μM) to LDHA are shown. (C) Intracellular and extracellular lactate assay were performed with cells incubated with 5 or 10 μM of berberine for 48 hours. The graph represents the relative intensity of lactate detected intracellularly and in the culture supernatant normalized per cell number compared to the respective controlled (NC) cells (*n* = 3 per group, *P < 0.05, **P < 0.01). (D) Control Panc02 and Panc‐1 cells were incubated with 5 or 10 μM of berberine (BBR) for 48 hours. The graph represents the relative protein expression ration of pAMPKa/AMPKa and LDHA normalized to *beta‐actin* compared to the nontreated control cells (*n* = 3 per group, *P < 0.05, **P < 0.01, ***P < 0.001). (E) MTT assay showed that berberine suppressed Panc02 and Panc‐1 pancreatic cancer cells proliferation and the cytotoxicities of berberine varies among different LDHA expression cells after 48 hours of incubation with berberine (*n *= 6 per group). (F) Colony formation assay was performed with controlled Panc02 and Panc‐1 cells treated with 5 μM berberine with or without supplementation with 10 mM L‐lactate. The graph represents the percentage of area covered by colonies after 10 days of incubation (*n* = 6 per group, **P < 0.01). (G) Boyden chamber invasion assay was performed for 24 hours with controlled Panc02 and Panc‐1cells treated with 5 μM berberine with or without supplementation with 10 mM L‐lactate. The graph represents the percentage of area covered by migrated cells after incubation (*n* = 6 per group, **P < 0.01). (H) Controlled Panc02 and Panc‐1 cells were incubated with or without 5 μM of berberine and supplanted with or without 10 mM L‐lactate for 24 hours. The graph represents the relative protein expression ration of pAMPKa/AMPKa and LDHA normalized to *beta‐actin* compared to the nontreated control cells (*n* = 3 per group, *P < 0.05, **P < 0.01, ***P < 0.001). BBR: berberine; IC50: half maximal inhibitory concentration; NC: controlled cells; SH: LDHA knockdown cells; OE: LDHA overexpressed cells

Observing LDHA overexpression inhibited AMPKa activation in pancreatic cancer cells, and knowing that berberine is a well‐reported AMPKa activator, we performed the molecular docking analysis on berberine and LDHA. The LDHA three‐dimensional structure was obtained and modeled using the known structure of human LDHA (PBD IDL1I10). Through constructing a structural model of LDHA with berberine, it was predicted that berberine binds to LDHA (Figure 5A). Berberine showed a FullFitness of −1292.49 kcal/mol and an estimated Δ*G* of −7.32 kcal/mol for the most favorable interaction with LDHA. Furthermore, surface plasmon resonance (SPR) biosensor analysis was performed to investigate the binding ability of LDHA to berberine. The results illustrated that berberine could specifically bind to LDHA (*K_d_* = 4.590e^−6^ M) (Figure 5B). Moreover, berberine treatment significantly suppressed intracellular lactate content at 5 μΜ and 10 μΜ (*P* < 0.01 and *P* < 0.001, respectively) and L‐lactate release in both Panc‐1 and Panc02 cell lines (*P* < 0.05 and *P* < 0.01, respectively) (Figure 5C), suggesting berberine as a functional inhibitor of LDHA (Figure S7). Furthermore, berberine dose‐dependently suppressed AMPKa activation and LDHA expression (Figure 5D) (*P* < 0.001). To further examine berberine's effect on LDHA expression, we performed in vitro cytotoxic assay of berberine on LDHA OE and SH pancreatic cancer cells. It was observed that berberine potently suppressed LDHA‐OE pancreatic cancer cell growth. We identified half‐maximal inhibitory concentration (IC50) approximately equal to 62.00 μM at 48‐hour treatment in LDHA‐OE Panc‐1 cells, and equal to 33.01 μM at 48‐hour treatment in LDHA knockdown Panc‐1 cells. The IC50 of Panc02 cells at 48‐hour treatment for SH and OE cells was 33.72 μM and 62.87 μM, respectively (Figure 5E). Functionally, exposure of L‐lactate to berberine‐treated cancer cells attenuated the growth and invasion suppression by berberine (Figure 5F and G), suggesting the restoration of LDHA attenuated the suppressive effect of berberine on PAAD. Interestingly, the AMPKa signaling activated by berberine was attenuated in the presence of L‐lactate (Figure 5H). These results indicate that berberine binding to LDHA suppressed LDHA/AMPKa signaling associated with pancreatic cancer cell progression. It should be noted that although LDHA inhibition plays a critical role in the pancreatic cancer inhibition effect of berberine, it is not the sole mechanism. Berberine also suppresses mitochondrial membrane potential in the treated PAAD cells (Figure S8), indicating other mechanisms shall be considered.

### 3.7 Oral administration of berberine suppressed proliferation and metastasis of pancreatic cancer in vivo

**FIGURE 6:**
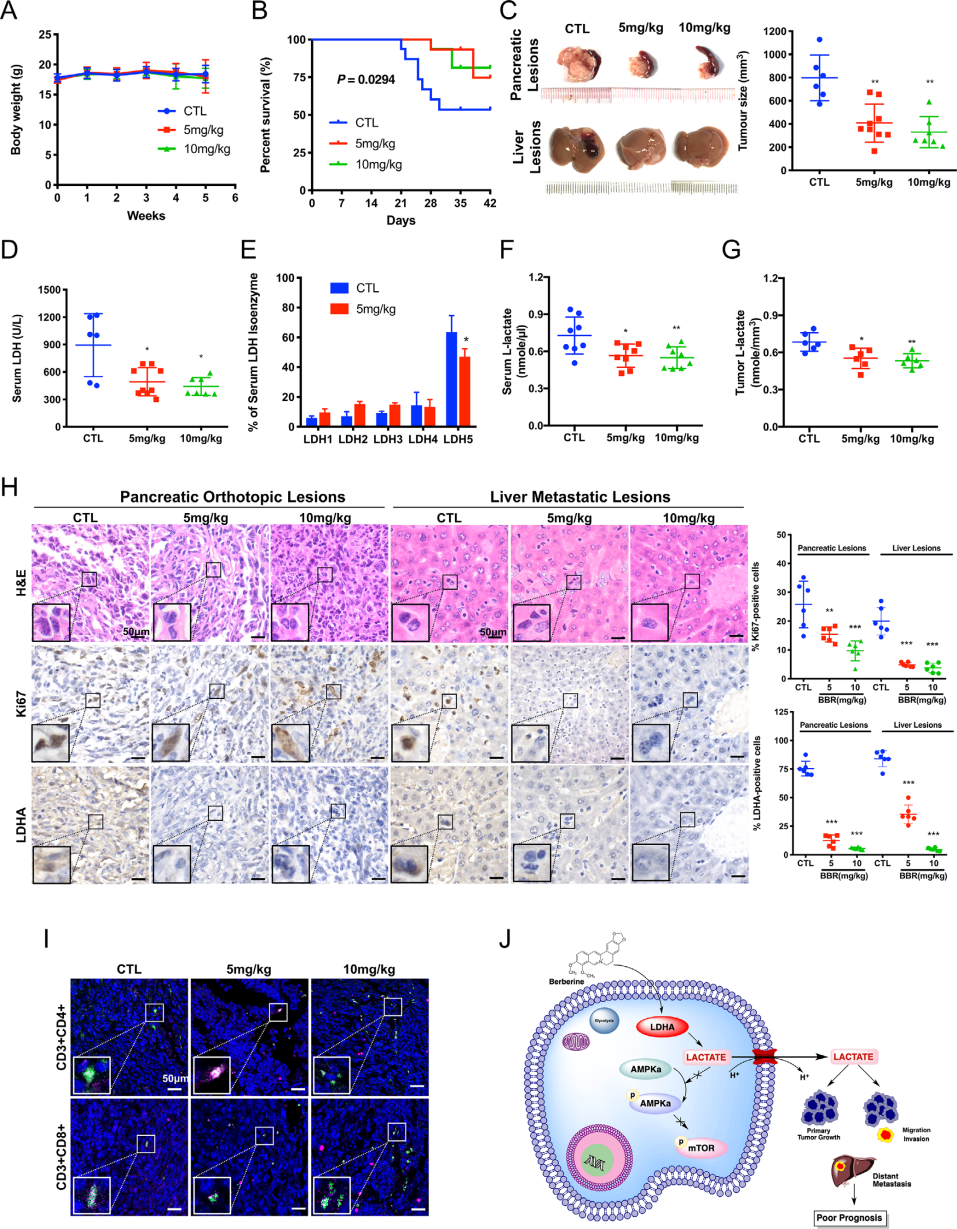
Berberine exhibited anti‐tumor effect in orthotopic pancreatic cancer implantation model. (A) Oral administration of berberine had minimal effect on the body weight of C57BL/6N mice. (B) Compared to the control (CTL, *n* = 16) group, berberine (5 mg/kg, *n* = 16 and 10 mg/kg, *n* = 16) can prolong survival in pancreatic cancer model (*P* = 0.0294). (C) Representative images of the dissected pancreas and liver at the end of berberine treatment showing that berberine intervention can regress orthotopic growth of implanted pancreatic cancer and suppress liver metastasis. The graph represents the orthotopic tumor size of the controlled group mice (CTL, *n* = 6), 5 mg/kg berberine group (*n* = 9) and 10 mg/kg berberine group (*n *= 7) by the end of the 4‐week intervention (**P < 0.01). (D) Compared to the control group, berberine treatment (5 mg/kg, *n* = 9 and10 mg/kg, *n* = 7) suppresses serum LDH level (*P < 0.05). (E) a shift of serum LDH isoenzyme to LDH‐5 was found and berberine treatment group exhibit lower serum LDH‐5 than that of the control (*P < 0.05). (F) Compared to the control (CTL, *n* = 6) group, berberine (5 mg/kg, *n* = 6, and 10 mg/kg, *n* = 6) treatment suppresses serum L‐lactate level (*P < 0.05, **P < 0.01) in murine in orthotopic pancreatic cancer implantation model. (G) Compared to the control (CTL) group, berberine (5 mg/kg and 10 mg/kg) treatment suppresses intratumoral L‐lactate level (*n* = 6 each group, **P < 0.01) in murine in orthotopic pancreatic cancer implantation model. (H) Representative histological and immunohistochemistry (IHC) photomicrographs of the tumor in individual groups. Paraffin‐embedded tumor tissues were stained with hematoxylin and eosin (H&E), anti‐Ki67 antibody and anti‐LDHA antibody to determine the pathological type, proliferation, and LDHA expression of the tumor cells upon different treatments. The graph represents the percentage of Ki‐67 and LDHA‐positive cells of pancreatic and liver lesions in control group (CTL, *n* = 6), 5 mg/kg berberine group (*n* = 6) and 10 mg/kg berberine group (*n* = 6) (**P < 0.01, ***P < 0.001). (I) Representative histological and immunofluorescent (IF) photomicrographs of the pancreatic orthotopic lesions. Paraffin‐embedded tumor tissues were stained with DAPI (Blue), anti‐CD3 (Red), anti‐CD4 (Green), and anti‐CD8 (Green) antibody in individual groups upon different treatments. (J) The schematic representative of regulatory mechanism underlying LDHA‐mediated inhibition of pancreatic cancer by berberine. CTL: control group; LDH: lactate dehydrogenase; HE: hematoxylin and eosin staining; LDHA: lactate dehydrogenase A

To further examine the effect of berberine in PAAD, we established pancreatic cancer orthotopic murine model in C57BL/6N mice by orthotopically injecting Panc02 cells into the head of the pancreas. Bodyweight measurements revealed that oral administration of berberine (5  and 10 mg/kg/2 days) had minimal effect on the body weight, indicating that berberine had no observational toxicity to the mice (Figure 6A). Survival analyses revealed that berberine prolonged mice survival in the pancreatic cancer model (*P* < 0.05) (Figure 6B). By the end of the experiment, it was found that mice receiving vehicle had enlarged pancreatic tumor and the presence of hepatic metastatic lesions, while the mice that received berberine treatment presented with a smaller pancreatic tumor and no hepatic lesion on the surface of the liver (Figure 6C). The reduction of pancreatic tumor size between vehicle and berberine‐treated groups was statistically significant (*P* < 0.01). Serum analysis revealed that, compared to the control group, berberine treatment suppressed serum LDH level (*P* < 0.05) (Figure 6D). Further, the berberine treatment group exhibited lower serum LDH‐5 than that of the control (*P* < 0.05) (Figure 6E). Moreover, berberine treatment significantly suppresses serum L‐lactate levels in both the serum (Figure 6G) and tumor (Figure 6F) of the orthotopic pancreatic cancer implantation model. Further immunostaining showed that berberine potently decreased LDHA expression in PAAD (Figure 6H). In addition, H&E staining revealed adenocarcinoma lesion of the pancreas and metastasis of tumor cells on the tissue of the vehicle group (Figure 6H). A decrease in proliferation in berberine‐treated mice liver and tumor tissues compared to that of the vehicle control was also observed by detecting the expression level of Ki67, a marker of cell proliferation (Figure 6H). Moreover, considering immunocompetent C57BL/6N mice were used in the present study, we further partially explored whether tumor‐infiltrating T cells play a role in tumor growth of the inhibition effect of berberine. Our results preliminarily showed that berberine treatment induces the number of T cells in the pancreatic tumors (Figures 6I and S9). Together, these results suggest that berberine suppressed tumor progression of PAAD in vivo through functional inhibition of LDHA (Figure 6J).

## 4 DISCUSSION

At present, despite considerable efforts to improve chemotherapy, the treatment outcome remains unsatisfactory for pancreatic adenocarcinoma. It is of value to develop simple and commonly used clinical parameters to predict the risk of tumor metastasis, overall prognosis and allow tailored treatment for individual patients. Although various studies suggested that LDH could indicate malignancies’ prognosis and is one of the risk factors in the International Prognostic Index for aggressive lymphoma and osteosarcoma. In PAAD, a meta‐analysis of 18 publications involving 3345 patients suggested that serum LDH level was significantly associated with worse overall survival; however, significant heterogeneities remain upon subgroup stratification by ethnicity, sample size, disease status, and survival analysis method and treatment.^22^ To our knowledge, no differences in the specific subtypes of LDH have been investigated. Furthermore, a previous study found that only 29% of colorectal cancer patients with LDHA overexpression had elevated serum LDH levels.^23^ In comparison, approximately 71% of patients with LDHA overexpression levels showed normal serum LDH levels,^23^ masking the diagnostic accuracy on serum LDH. Our study demonstrated for the first time that LDH‐5, encoded by the LDHA gene, exerts better prognostic value than serum LDH in pancreatic cancer and may complement tissue LDHA levels in providing prognosis information for patients with unresectable pancreatic cancer, where obtaining tissue biopsy may be difficult.

L‐lactate overproduction, as the end product of glycolysis, reflects aberrant tumor glucose metabolism and may also serve as a metabolic derangement marker. Elevated tumor L‐lactate concentrations have been linked with increased risk of subsequent development of metastases in head‐and‐neck cancer patients.^24^ However, tumor L‐lactate concentration measurements are limited in pancreatic cancer patients. The technique requires fresh‐frozen tumor samples obtained by biopsy of the primary site,^24^ which may be complicated with acute pancreatitis and pancreatic fine‐needle aspiration is common practice clinically. As for serum lactate, a previous study failed to determine the correlation between the risk of metastasis with maximum serum lactate levels in patients with lung cancer. Nevertheless, the study identified that elevated lactate (1.4 mmol/L) was associated with significantly shorter overall survival from days to weeks.^25^ This may be due to the high comorbidity of critical conditions such as hyperlactatemia and acidosis in patients related to not only a sole tumor‐related cause but also oxygenation imbalance. Our study investigated the baseline serum LDH and LDH‐5, which may better outline the long‐term overall survival, allow tailoring treatment for patients. It may be a surrogate to monitor targeted inhibition of LDHA.

Currently, several LDHA inhibitors have been proposed and are mainly involved in the following categories: pyruvate‐competitive, NADH‐competitive, pyruvate and NADH‐competitive, free enzyme‐binding inhibitor, and others. Oxamate, ss a pyruvate analog, competes with LDHA substrate, and its function as an LDHA inhibitor has been extensively verified in vitro. However, the effective dose of oxamate in vitro is too high for in vivo administration due to limited membrane permeability.^26^, ^27^ Gossypol, a natural phenol derived from cotton plants, is another LDHA inhibitor that targets LDHA by competing with NADH and promising antiproliferative effect.^28^, ^29^, ^30^ However, due to the potential interaction of gossypol with different cellular components of biological functions, the non‐specific toxicity of gossypol has made further translational attempts unsuccessful.^28^, ^31^ Research on other LDHA inhibitors, such as FX11, Quinoline 3‐sulfonamides, NHI, PSTMB, and Galloflavin, remain mostly in vitro and are similarly drawback by their nonspecific toxicity, low renal clearance, or incompatible with oral bioavailability.^32^, ^33^, ^34^, ^35^ Therefore, the development of a safe and effective novel LDHA inhibitor with clinically achievable dosage remains necessary.

Berberine is a quaternary ammonium salt from the protoberberine group of isoquinoline alkaloids derived from Coptidis Rhizoma. As an over‐the‐counter (OTC) drug approved by the Chinese Food and Drug Administration (CFDA), berberine is available to treat microbial‐dependent or ‐independent gastrointestinal disorders. Over decades, scanty case of any undesirable effects has been reported^36^ and berberine at a dose of 1.0‐1.5 g daily for three consecutive months is safe in multiple clinical trials including Asian adults.^37^ In the present study, we demonstrated that berberine significantly reduced pancreatic cancer proliferation and invasion in vitro at a dose as low as 5 μM and suppresses tumor growth and metastasis at 5 mg/kg in vivo, equivalent to that of approximately 25 mg daily for a 70 kg adult.^38^ However, berberine had been reported to have a poor pharmacokinetic profile, and the future application may require combination administration, novel formulation technology, and derivatization.^39^ The tumor suppression effect of berberine may also be the consequence of mitochondria inhibition through AMPK activation,^40^, ^41^, ^42^ apoptosis and autophagy induction,^43^, ^44^, ^45^ oxidative phosphorylation, and redox regulation^46^, ^47^; thereby, although LDHA inhibition plays a critical role, this is not the sole mechanism of berberine on suppressing pancreatic cancer cell progression. Furthermore, berberine was previously reported as a mitochondrial respiration inhibitor and can suppress mitochondrial complex I function in diabetic murine models^48^; therefore, it may be expected that berberine induces lactic acidosis.^48^, ^49^, ^50^ On the contrary, our study in pancreatic cancer models showed that berberine inhibited LDHA expression, enzymatic activity, and lactate content in both murine serum and orthotopic tumors and prolonged survival in pancreatic cancer models. However, a more extended observation period for survival data, further investigation into the role of LDHA‐dependent effect on tumor‐infiltrating T cells, and evaluating the precise mechanism of berberine in regulating pancreatic cancer remains to be elucidated.

Cancer cells possess distinct metabolism to sustain the energy need and provide sufficient biomaterials for rapid proliferation and metastasis.^51^ As the by‐product of accelerated glycolysis, lactate accumulation in the tumor microenvironment induces tumor escape from immune surveillance by T and natural killer cells and promotes tumor proliferation.^52^ Furthermore, lactate induces tumor‐associated‐macrophage polarization into the M2‐like phenotype to resolve inflammation in Lewis lung carcinoma (LLC) and B16‐F1 (B16) melanoma murine models.^53^ On the other hand, lactate accumulation is linked to chronic inflammatory microenvironment. Previous studies suggested that the expression of SLC5A12, a low‐affinity sodium‐coupled lactate transporter, by CD4+ T cell subset promotes lactate uptake and further supports pro‐inflammatory response by the CD4+ T cells.^54^, ^55^

The high‐energy demands of cancer cells had been purposed to consequently increase in L‐lactate production without an oxidative phosphorylation.^56^ The L‐lactate‐rich microenvironment subsequently induced cellular metabolomic adaptation, along with the attempt to reestablish glycolytic flux and mitochondrial oxidation, generated macromolecules favoring cell division, reactive oxygen species (ROS) signaling, genomic instabilities, and cancer progression.^57^, ^58^, ^59^, ^60^ Furthermore, the elevated catalytic efficiency of glycolytic enzyme promoted L‐lactate secretion and formed an autocrine lactate loop promoting malignant transformation.^57^, ^61^, ^62^ The possible role of l‐lactate metabolism and transport in cancer biochemistry has long been ignored in cancer research^63^ and future research is warranted.

Furthermore, lactate may also serve as a carbon source in tricarboxylic acid (TCA) cycle, and tumor cells exhibit autonomous lactate uptake to complement glucose metabolism in human nonsmall‐cell lung cancers(NSCLC).^64^ It is worth noting that Kras‐mutant NSCLC showed heterogenous lactate labeling compared to the adjacent lung, highlighting that heterogenous lactate metabolism may be associated with Kras mutation.^64^ In pancreatic cancer, the signature genetic mutational landscape of over 90% of Kras oncogene mutations plays a critical role in controlling tumor metabolism by enhancing glucose uptake, accelerating glycolytic flux, and increasing lactate production and fuelling cancer progression.^65^, ^66^ Previous studies investigating the mRNA expression profiling of doxycycline‐induced oncogenic Kras expression showed that oncogenic Kras contributes to enhanced glycolytic flux by transcriptionally upregulating LDHA expression.^66^ Additionally, the metabolomic changes were highly concordant with transcriptional changes, such as oncogenic Kras extinction, leading to decreased lactate production, suggesting oncogenic Kras regulates glycolysis rate limiting enzyme LDHA and is essential for glucose utilization.^66^ Furthermore, as PAAD exhibits characteristically dense stromal and low vascular density,^67^ whether and how the end product of glycolysis, lactate, alters immune cell function in this unique tumor microenvironment warrants further studies.

In the present study, we have investigated the LDHA/AMPK‐mTOR axis, and we speculate that LDHA might also regulate by other pathways and transcription factors. It is possible that the LDHA overexpression promoted L‐lactate production, and increased L‐lactate as characterized by L‐lactate production further suppresses AMPK activation. Via the inducing L‐lactate production of LDHA overexpressed cells, extracellular acidification may play crucial roles in tumor proliferation, angiogenesis, the epithelial‐to‐mesenchymal transition, local invasion, and the formation of distant metastases. The role of LDHA/LDH5 and lactate transporters (eg, the MCT family) and the pathway associated with glucose metabolism, energy homeostasis, hypoxia, and tumor microenvironment requires further investigations until the development of specific inhibitors for clinical use.

## 5 CONCLUSIONS

In summary, based on the in vitro and in vivo functional studies, our study demonstrated that LDHA is overexpressed in PAAD and is associated with PAAD progression. Our clinical and mechanistic findings indicated that LDHA overexpression leads to increased pancreatic cancer growth and metastasis, and vice versa. LDHA overexpression reduced the phosphorylation of metabolic regulator AMPK and promoted the downstream mTOR phosphorylation in PAAD cells. Inhibition of mTOR repressed the LDHA‐induced PAAD cell proliferation and invasion. This study identifies a novel biomarker that can be detected in noninvasive means for identifying patient subgroup for treatment, and found a new therapeutic agent for controlling pancreatic cancer. We found that the functional inhibition of LDHA by a natural product, berberine, reduced activity, and protein expression in PAAD cells at a clinically achievable dose. Berberine inhibited the in vitro proliferation and invasion of PAAD cells and suppressed tumor progression in vivo. The restoration of LDHA attenuated the suppressive effect of berberine on PAAD. Together, our findings suggest that LDHA may be a potential therapeutic target in the treatment of human PAAD.

## CONFLICTS OF INTEREST

The authors declare that they have no potential conflicts of interest.

## ETHICS DECLARATIONS

This study has been approved by the ethics committee of Fudan University Shanghai Cancer Center. All procedures were performed in accordance with the ethical standards of the institutional guidelines and with the 1964 Helsinki declaration and its later amendments. Written informed consent obtained from individual patients according to institutional guidelines. The datasets generated during and/or analyzed during the current study are available from the corresponding author on reasonable request.

## AUTHOR CONTRIBUTIONS

Conception and design: Y. Feng and Z. Chen. Development of methodology: C.‐S. Cheng, H.‐Y Tan, and N. Wang. Acquisition of data (provided animals, acquired and managed patients, provided facilities, etc.): C.‐S. Cheng, H.‐Y Tan, N. Wang, L. Chen, Z. Meng, Z. Chen, and Y. Feng. Analysis and interpretation of data (eg, statistical analysis, biostatistics, computational analysis): C.‐S. Cheng, H.‐Y Tan, and N. Wang. Writing, review, and/or revision of the manuscript: C.‐S. Cheng, H.‐Y Tan, N. Wang, L. Chen, Z. Meng, Z. Chen, and Y. Feng. Administrative, technical, or material support (ie, reporting or organizing data, constructing databases): C.‐S. Cheng, H.‐Y Tan, and N. Wang. Study supervision: Z. Chen and Y. Feng.

## Supporting information

Supporting Information 1

Supporting Information 1
