# Supplementary material for: Functional inhibition of lactate dehydrogenase suppresses pancreatic adenocarcinoma progression
Source: Clin Transl Med. 2021 Jun 28;11(6):e467. doi: 10.1002/ctm2.467 (PMC8238920; doi:10.1002/ctm2.467)
Supplement: Supplementary file 1 — Supporting Information 1 [file CTM2-11-e467-s001.docx]

Electronic Supplementary Information


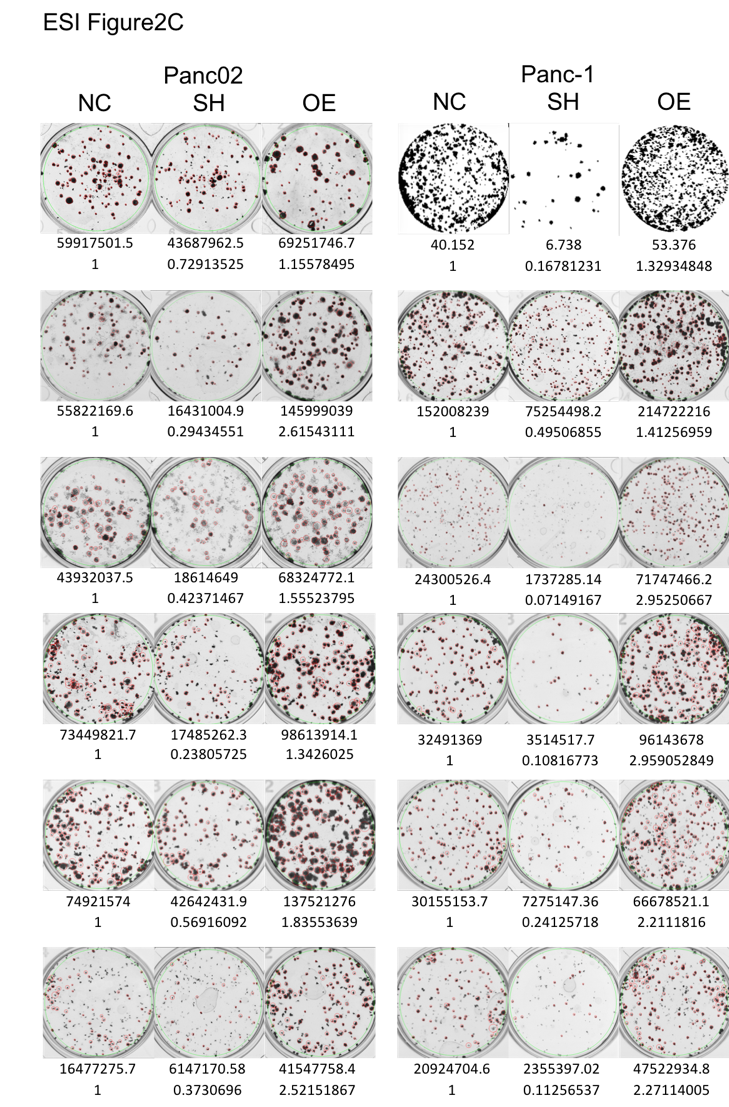


ESI Figure 2C. Figures obtained and counts collected for the quantification and statistical analysis of the controlled (NC), LDHA knockdown (SH), and LDHA overexpression (OE) cells in Panc-1 and Panc02 cells.


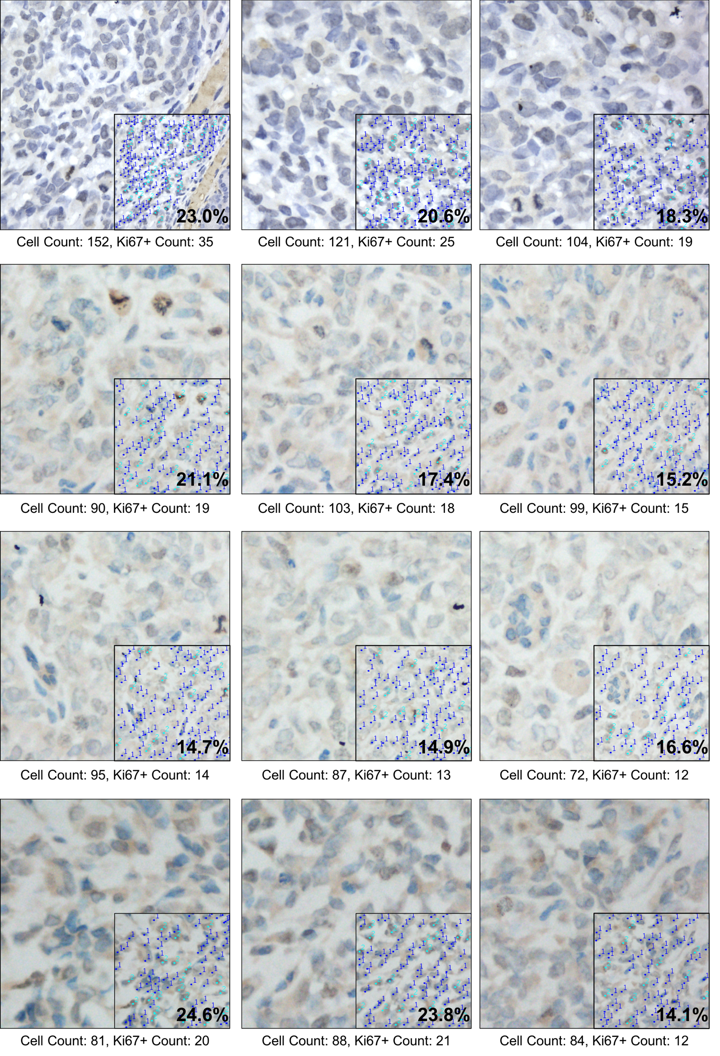


ESI Figure 2F. Figures obtained and counts collected for the quantification and statistical analysis of the Ki67-positive cell percentage in the histological staining (IHC) of the LDHA-overexpressing cell-derived tumor tissue.


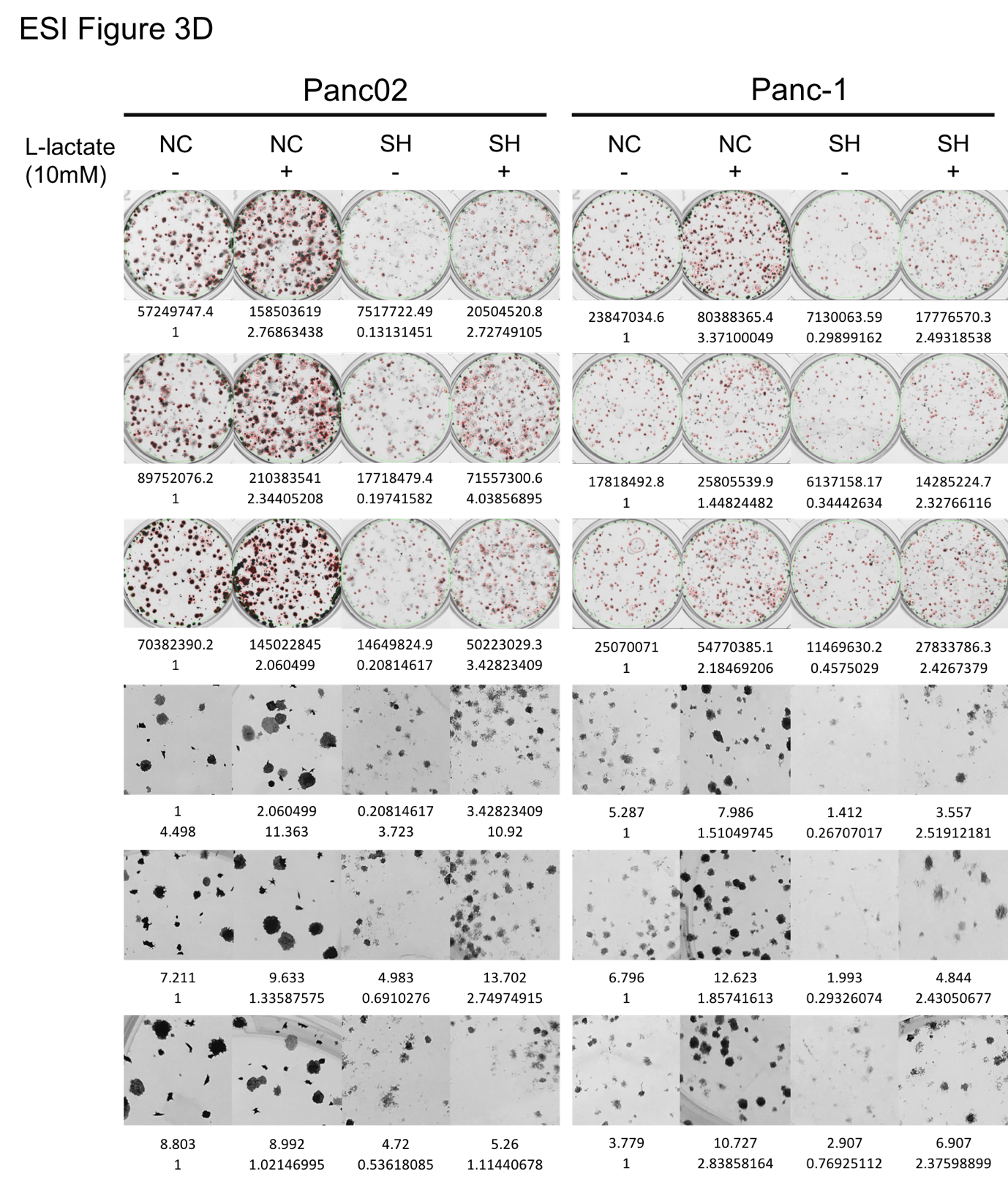


ESI Figure 3D. Figures obtained and counts collected for the quantification and statistical analysis of the Panc-1 and Panc02 LDHA control (NC) and knockdown (SH) cells with or without L-lactate


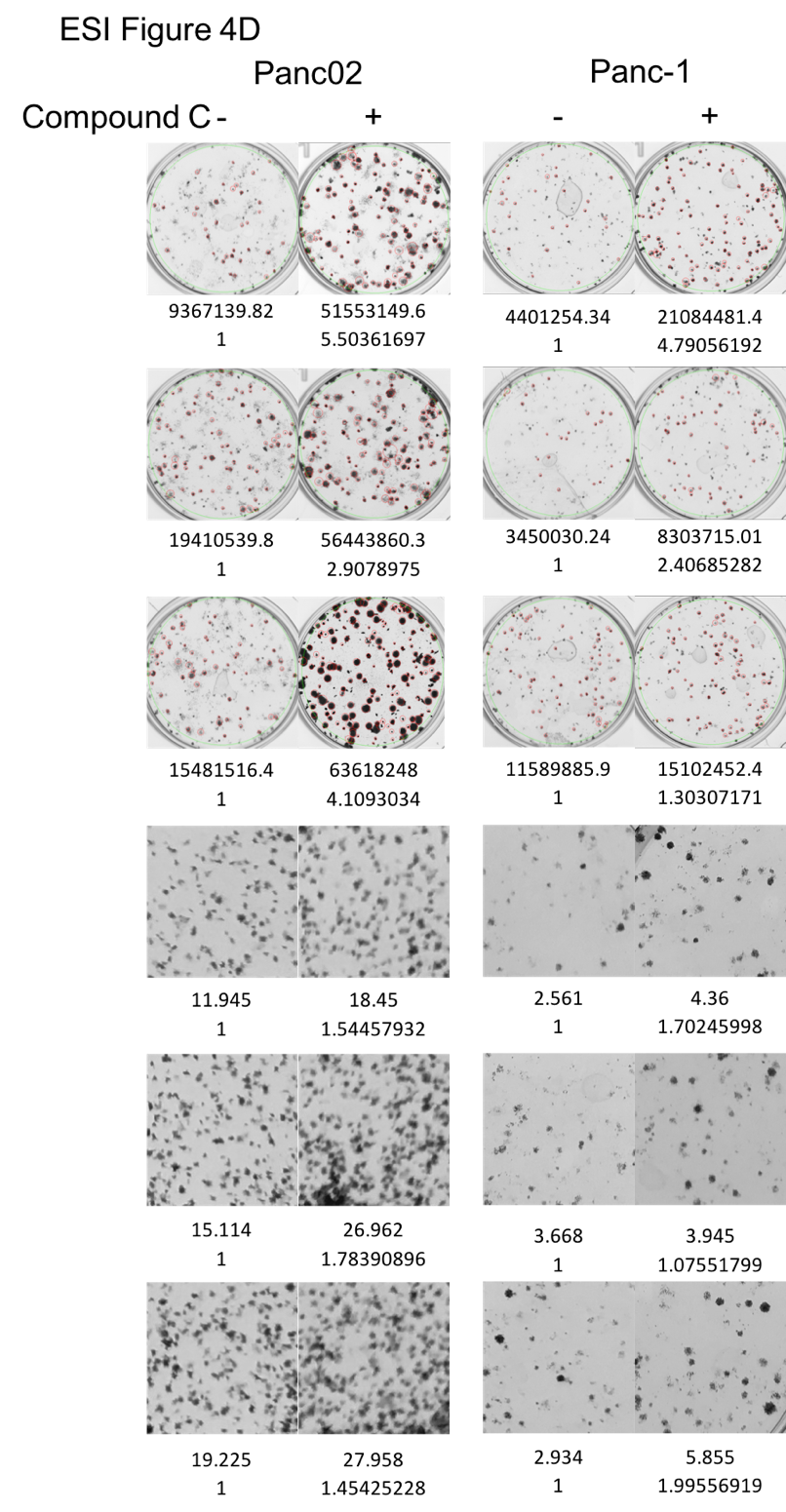


ESI Figure 4D. Figures obtained and counts collected for the quantification and statistical analysis of the Panc-1 and Panc02 LDHA knockdown (SH) cells with or without Compound C.


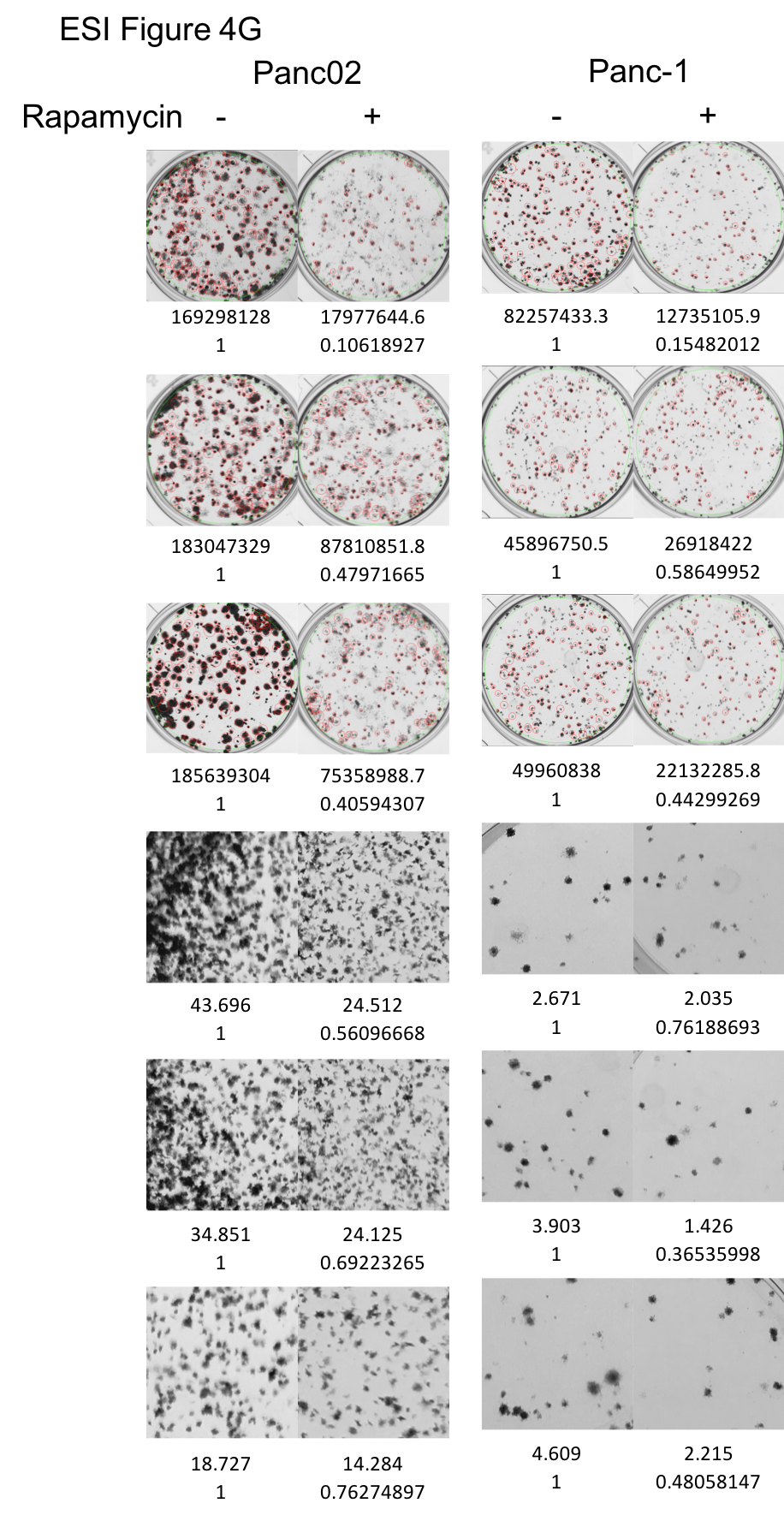


ESI Figure 4G. Figures obtained and counts collected for the quantification and statistical analysis of the Panc-1 and Panc02 LDHA overexpressed (OE) cells with or without Rapamycin.


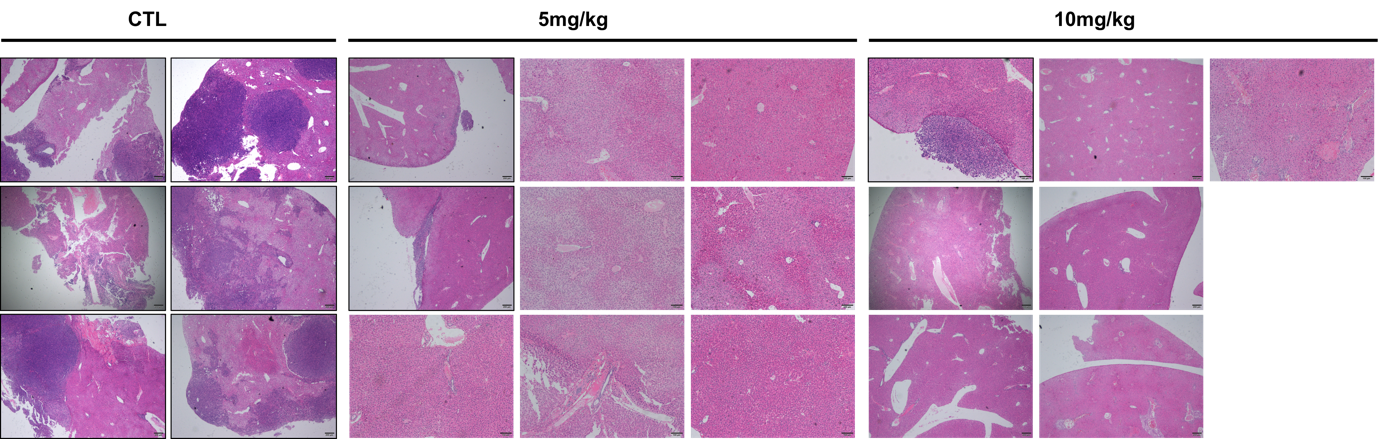


ESI Figure 6C. Representing figures obtained for the liver serial H&E staining of the surviving mice in the CTL, berberine 5mg/kg and 10mg/kg groups.
