# Supplementary material for: Functional inhibition of lactate dehydrogenase suppresses pancreatic adenocarcinoma progression
Source: Clin Transl Med. 2021 Jun 28;11(6):e467. doi: 10.1002/ctm2.467 (PMC8238920; doi:10.1002/ctm2.467)

**Table S1.** Detailed information about the antibodies used.

| Name | Brand (Catalog No.)  Source/Specificity | Reactivity# | Applications | Batch No. |
| --- | --- | --- | --- | --- |
| AMPKα (D5A2) | CST (#2603P)  Rabbit mAb | H M R Mk B | WB(1: 1000) | Ref:03/2013 Lot:10 |
| Phospho-AMPKα (Thr172) (40H9) | CST (#2535P)  Rabbit mAb | H M R Hm Mk Dm Sc | WB(1: 1000) | Ref:03/2013 Lot:16 |
| β-Actin (13E5) | CST (#4970S)  Rabbit mAb | H M R Mk Dm Z | WB(1: 1000) | Ref:11/2017 Lot:15 |
| Ki-67 | Abcam (ab15580)  Rabbit pAb | H M R Hm Mk Pg | IHC (1:200) | Lot:GR73724-1 |
| LDHA | Abcam (ab101562)  Rabbit mAb | H M R | WB(1: 1000)  IHC(1:200) | Lot:GR156108-1 |
| mTOR (7C10) | CST (#2983S)  Rabbit mAb | H M R Mk | WB(1: 1000) | Ref:03/2017 Lot:14 |
| Phospho-mTOR (Ser2448) (D9C2) | CST (#5536S)  Rabbit mAb | H M R Mk | WB(1: 1000) | Ref:08/2019 Lot:9 |

#Specified by the manufacturer; WB: Immunoblotting; IHC: Immunohistochemistry; H: Human; M: Mouse; R: Rat; Hm: Hamster; Mk: Monkey; Mi: Mink; Dm: D. melanogaster; Z: Zebrafish; B: Bovine; Pg: Pig; Sc: S. cerevisiae.

**Table S2.** Significant changes of LDHA expression in transcription level between Pancreatic Cancer and Non-cancerous pancreatic tissues (ONCOMINE).

| **Types of Cancer v.s. Non-cancerous** | **Over‑expression Gene Rank** | **Fold Change** | ***p*-value** | **t-test** | **Number of Samples** | **Number of Measured genes** | **Platform** | **Ref** |
| --- | --- | --- | --- | --- | --- | --- | --- | --- |
| Pancreatic Adenocarcinoma v.s. Pancreatitis | 205 (in top 4%) | 1.918 | 9.74E-4 | 5.147 | 27 | 5,338 | HumanGeneFL Array | ^48^ |
| Pancreatic Carcinoma v.s. Pancreas | 311 (in top 3%) | 3.912 | 1.30E-4 | 4.750 | 17 | 12,624 | Human Genome U133A Array | ^49^ |
| Pancreatic Ductal Adenocarcinoma v.s. Pancreatic Intraepithelial Neoplasm | 929 (in top 6%) | 1.700 | 0.004 | 3.211 | 38 | 15,741 | Platform not pre-defined in Oncomine | ^50^ |
| Pancreatic Carcinoma v.s. Pancreas | 1317 (in top 7%) | 2.436 | 4.33E-5 | 4.882 | 52 | 19,574 | Human Genome U133 Plus 2.0 Array | ^51^ |
| Pancreatic Ductal Adenocarcinoma v.s. Pancreas | 1764 (in top 10%) | 2.463 | 4.39E-8 | 6.196 | 78 | 19,574 | Human Genome U133 Plus 2.0 Array | ^52^ |

**Supplementary Figures**

**Figure S1.** LDHAexpression levels in pancreatic cancers. **(A)** Expression profile of LDHA, LDHB, and LDHC between human pancreatic tumour tissues and non-tumour normal tissue in dataset GDS1403 and GDS4336. **(B)** Kaplan-Meier plots of overall and disease-free survival in pancreatic cancer patients with LDHA, LDHB and LDHC expressions. Higher mRNA expression of LDHA was associated with poorer overall survival and disease free survival in pancreatic cancer patients (*p* < 0.0001, *p* < 0.0001, respectively). There are no significant correlation in LDHB or LDHC expression with patients’ overall and disease-free survival rate.


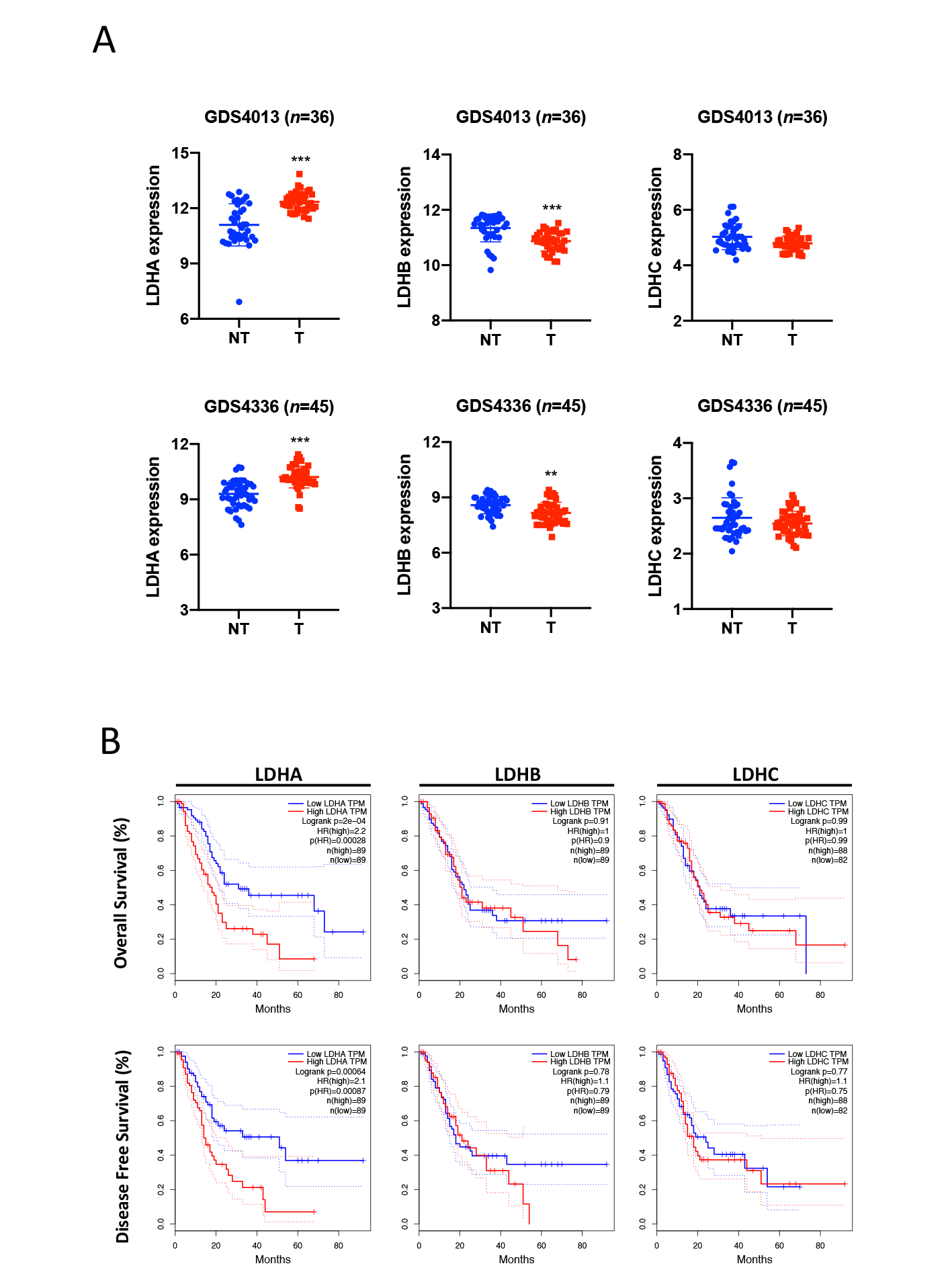


**Figure S2.** LDHA overexpression promoted liver metastasis in the LDHA-OE Panc-1 models. Serial H&E staining identified adenocarcinoma in the liver section of the LDHA-OE group (black arrowed).


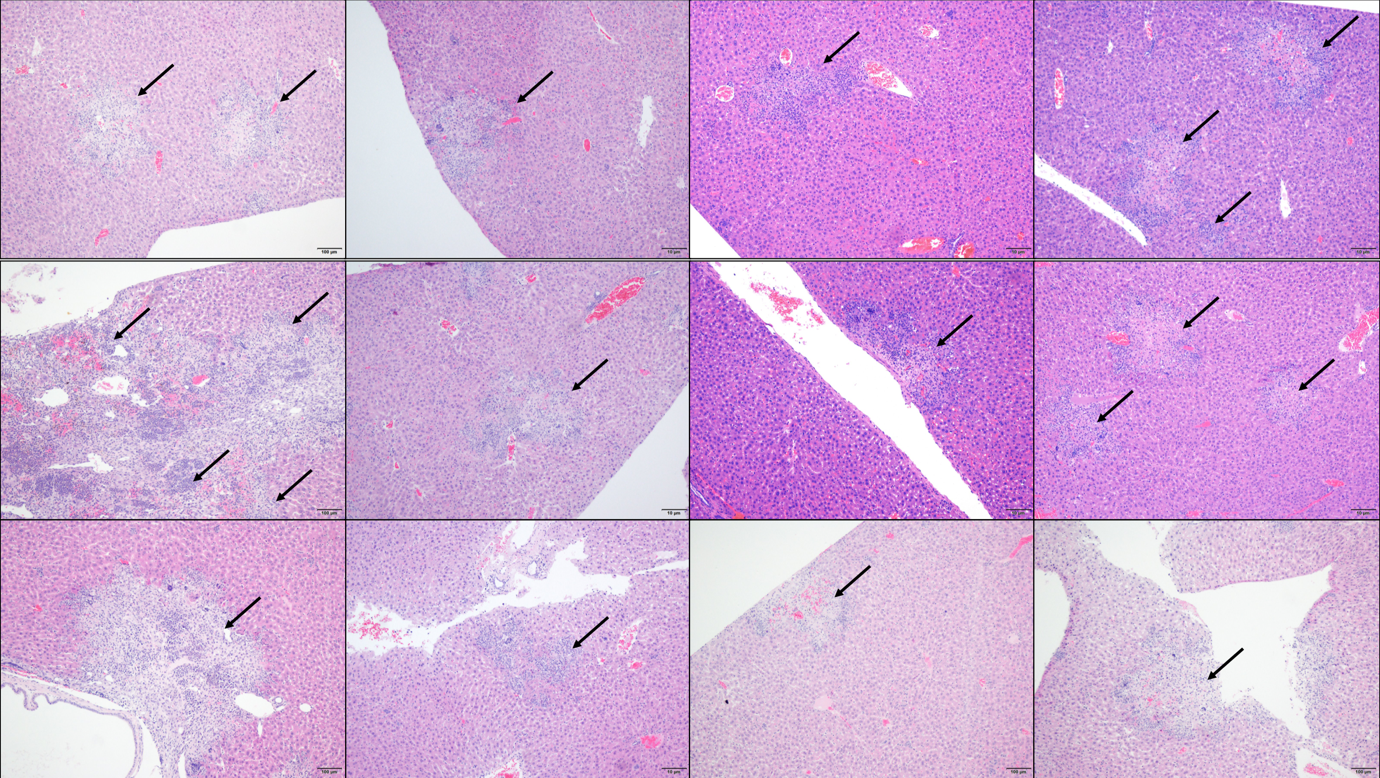


**Figure S3.** LDHA knockdown abolished the tumorigenesis and liver metastasis capabilities of pancreatic cancer cells. **(A)** The macroscopic view of orthotopic implantation model with LDHA-SH panc-1 cells (*n* = 6 per group). No obvious abnormal cells was observed in the serial histological sectioning and H&E staining of the pancreatic tissues. **(B)** The macroscopic view of liver metastasis model with LDHA-SH panc-1 cells (*n* = 6 per group). No obvious abnormal cells was observed in the serial histological sectioning and H&E staining of the hepatic tissues.


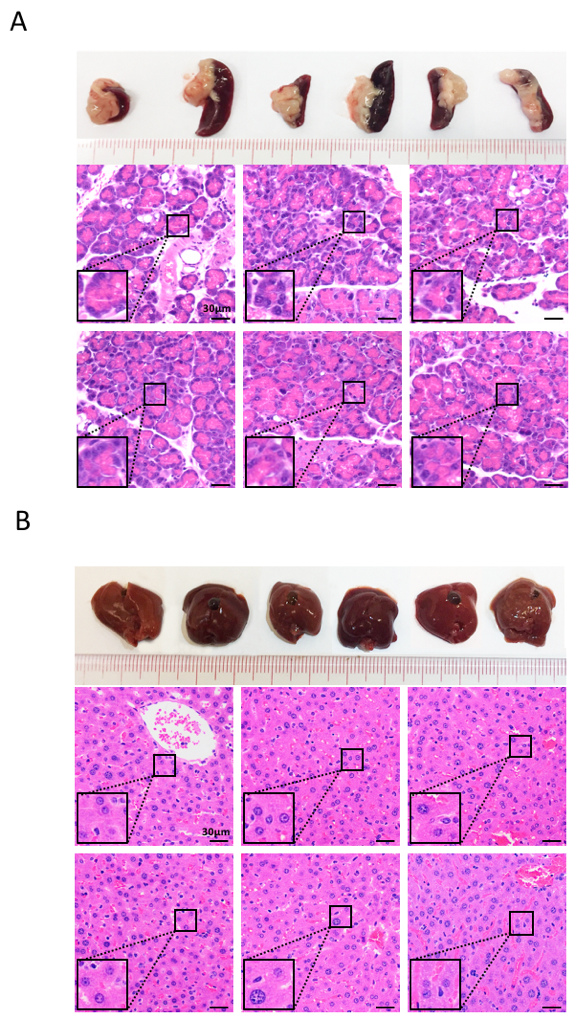


**Figure S4.** The cell viability of Panc02 and Panc-1 cells when exposed to increasing concentrations of L-lactate (6.25-50 mM) for 48 h.

**Figure S5.** Introduction of 7ACC2 to LDHA overexpressed (OE) cell suppressed the **(A)** clonogenic and **(B)** invasion capabilities of PAAD cells.

**
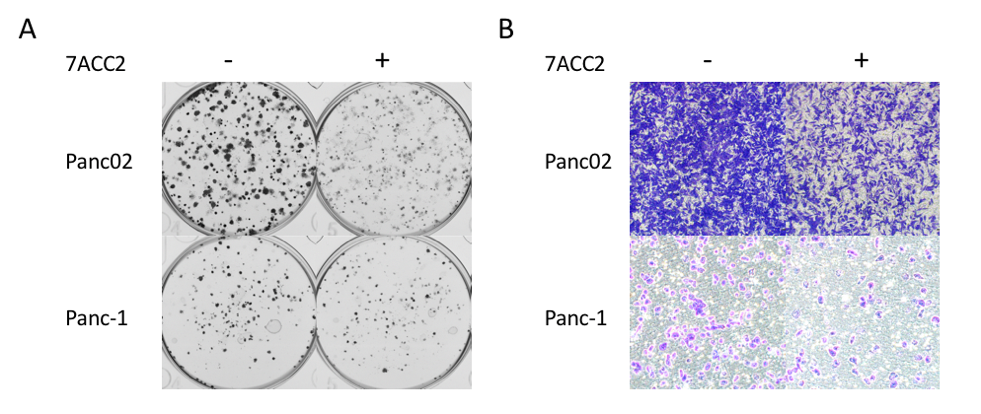
**

**Figure S6.** LDHA knockdown (SH) significantly increases cellular AMP/ATP ratio compared to the controlled (NC) cells (**p* < 0.05, *** *p* < 0.001).

**Figure S7.** Relative LDHA inhibition rate (*n* = 3) of potential inhibitors including FX-11, GSK2837808a, Gossypol, Berberine and DMSO was used as solvent control.

**Figure S8.** Effects of berberine on mitochondrial function in PAAD cells. Panc-1 and Panc02 cells exposed to berberine 5 μM or 10 μM for 24 h and the mitochondrial membrane potential was determined using JC-1 dye.

**
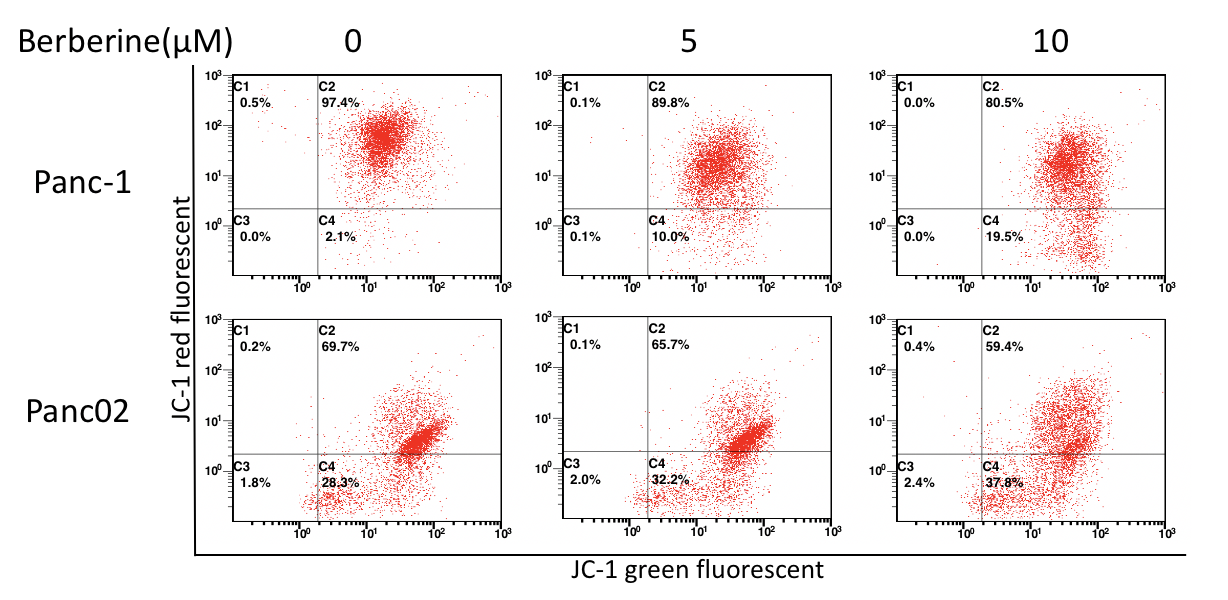
**

**Figure S9.** Berberine intervention shows a trend of enhancing tumour infiltrating T cells in C57BL/6N murine orthotopic pancreatic cancer implantation model. The graph represents the percentage of **(A)** CD3+CD4+, **(B)** CD3+CD8-positive cells and **(C)** relative CD4+/CD8+-positive cell ratio of the orthotopic pancreatic cancer in control group (CTL, *n* = 6), 5mg/kg berberine group (*n* = 6) and 10mg/kg berberine group (*n* = 6) (***p* < 0.01).


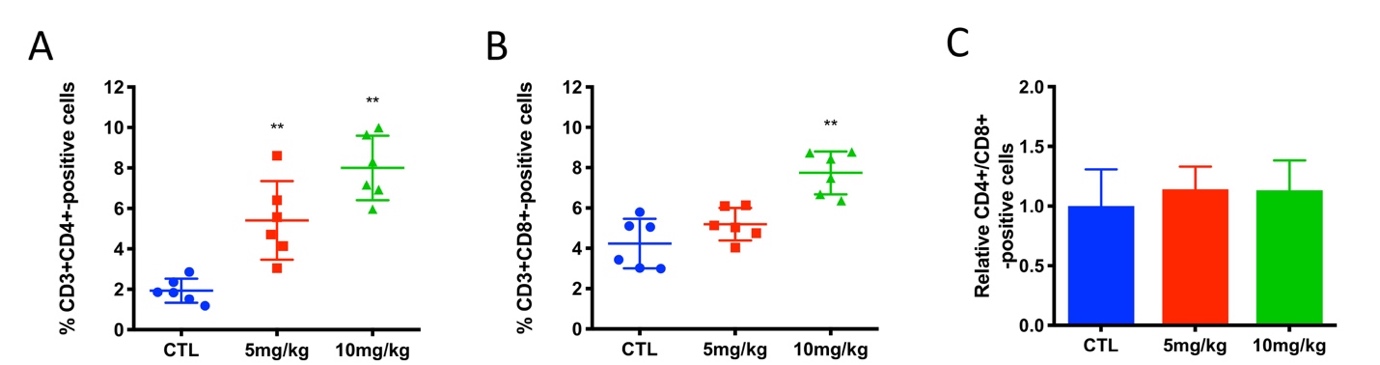

Supplement: Supplementary file 2 — Supporting Information 1 [file CTM2-11-e467-s002.docx]
